# Maternal characteristics associated with the dietary intake of nitrates, nitrites, and nitrosamines in women of child-bearing age: a cross-sectional study

**DOI:** 10.1186/1476-069X-9-10

**Published:** 2010-02-19

**Authors:** John S Griesenbeck, Jean D Brender, Joseph R Sharkey, Michelle D Steck, John C Huber, Antonio A Rene, Thomas J McDonald, Paul A Romitti, Mark A Canfield, Peter H Langlois, Lucina Suarez, the National Birth Defects Prevention Study

**Affiliations:** 1 Department of Epidemiology and Biostatistics, School of Rural Public Health, Texas A&M Health Science Center, College Station, TX, 77843-1266, USA; 2 Department of Social and Behavioral Health, School of Rural Public Health, Texas A&M Health Science Center, College Station, TX, 77843-1266, USA; 3 Department of Environmental and Occupational Health, School of Rural Public Health, Texas A&M Health Science Center, College Station, TX, 77843-1266, USA; 4 Department of Epidemiology, 200 Hawkins Dr, University of Iowa, Iowa City, IA, 52242, USA; 5 Birth Defects Epidemiology and Surveillance Branch, Texas Department of State Health Services, PO Box 149347, MC 1964, Austin, TX, 78714-9347, USA; 6 Environmental Epidemiology and Disease Registries Section, Texas Department of State Health Services, 1100 W. 49th St, Austin, TX, 78756, USA

## Abstract

**Background:**

Multiple *N*-nitroso compounds have been observed in animal studies to be both mutagenic and teratogenic. Human exposure to *N*-nitroso compounds and their precursors, nitrates and nitrites, can occur through exogenous sources, such as diet, drinking water, occupation, or environmental exposures, and through endogenous exposures resulting from the formation of *N*-nitroso compounds in the body. Very little information is available on intake of nitrates, nitrites, and nitrosamines and factors related to increased consumption of these compounds.

**Methods:**

Using survey and dietary intake information from control women (with deliveries of live births without major congenital malformations during 1997-2004) who participated in the National Birth Defects Prevention Study (NBDPS), we examined the relation between various maternal characteristics and intake of nitrates, nitrites, and nitrosamines from dietary sources. Estimated intake of these compounds was obtained from the Willet Food Frequency Questionnaire as adapted for the NBDPS. Multinomial logistic regression models were used to estimate odds ratios and 95% confidence intervals for the consumption of these compounds by self-reported race/ethnicity and other maternal characteristics.

**Results:**

Median intake per day for nitrates, nitrites, total nitrites (nitrites + 5% nitrates), and nitrosamines was estimated at 40.48 mg, 1.53 mg, 3.69 mg, and 0.472 μg respectively. With the lowest quartile of intake as the referent category and controlling for daily caloric intake, factors predicting intake of these compounds included maternal race/ethnicity, education, body mass index, household income, area of residence, folate intake, and percent of daily calories from dietary fat. Non-Hispanic White participants were less likely to consume nitrates, nitrites, and total nitrites per day, but more likely to consume dietary nitrosamines than other participants that participated in the NBDPS. Primary food sources of these compounds also varied by maternal race/ethnicity.

**Conclusions:**

Results of this study indicate that intake of nitrates, nitrites, and nitrosamines vary considerably by race/ethnicity, education, body mass index, and other characteristics. Further research is needed regarding how consumption of foods high in nitrosamines and *N*-nitroso precursors might relate to risk of adverse pregnancy outcomes and chronic diseases.

## Introduction

*N*-nitroso compounds are known to be potent carcinogens and are known to cause congenital malformations in animal models [1-3]. Multiple *N*-nitroso compounds have been observed in animal studies to be both mutagenic and teratogenic in nature [2-11]. Furthermore, these compounds have been associated with adverse reproductive outcomes and various types of cancers in humans [12-19]. Some studies have suggested that nitrosamines and *N*-nitroso precursors (nitrates and nitrites) may have an etiologic role in adverse reproductive outcomes in humans, including birth defects and spontaneous abortion [12,19].

Human exposure to *N*-nitroso compounds and their precursors (nitrates and nitrites) can occur through exogenous sources, such as diet, drinking water, occupation, or environmental exposures, and through endogenous exposures resulting from the formation of *N*-nitroso compounds in the body [18,20-24]. Nitrosamines and nitrosamides are the two groups of chemicals that comprise *N*-nitroso compounds. Both groups are characterized by a nitroso group (--N = O) attached to a nitrogen atom (--N--N = O) [25]. Generally, *N*-nitroso compounds can be formed by the reaction of a nitrite compound with amines or amides; this process is known as *N*-nitrosation [25]. Endogenous formation has been reported to account for up to 75 percent of the total nitrosamine exposure in humans [26]. In addition to dietary exposures, preformed nitrosamines have been found in beer and to a lesser extent, distilled spirits. Beer, presumably because of the malting process, contains volatile nitrosamines and has been implicated as a significant contributor to total dietary exposure of nitrosamines [26]. Ethanol has also been noted in several animal studies to increase internal exposure to nitrosamines by suppressing hepatic clearance of these compounds [27,28]. Nitrates and nitrites, under normal gastric conditions, are known to be precursors to *N*-nitroso compound formation in the presence of secondary or tertiary amines or amides [18]. Approximately 5% of ingested nitrates in food and water are converted to nitrite in the saliva, further promoting endogenous nitrosamine formation [1]. However, estimated dietary intake of nitrates may be 20-fold or higher than intake of nitrites, thereby contributing significant amounts of endogenous nitrite.

Although the formation of nitrosamines from nitrates may be important in the subsequent development of birth defects and cancer, there have been reported benefits of dietary nitrate consumption, especially from green, leafy vegetables. In addition to the vitamin and mineral content associated with vegetable consumption, dietary nitrates from vegetables have been described as a natural, low-cost approach for treatment of cardiovascular disease [29]. The bioactivation of dietary nitrates has been hypothesized as the mechanism of action converting consumed nitrates to the more vasoprotective nitric oxide which lowers blood pressure and may prevent endothelial dysfunction and platelet aggregation [29].

While results from studies have suggested both beneficial and adverse effects from exposure to nitrates and nitrites, very little is known about the demographic and behavioral factors related to the consumption of these compounds. Differences in risk for various adverse reproductive outcomes and chronic diseases might be due, in part, to low or high dietary consumption of nitrates, nitrites, and nitrosamines. Using food frequency data reported by control participants in the National Birth Defects Prevention Study (NBDPS) [30], we: 1) described the pattern of maternal dietary nitrate, nitrite, total nitrite, and nitrosamine consumption; 2) estimated consumption of these compounds by maternal race/ethnicity and other demographic and behavioral characteristics; and 3) examined the relative contributions of these compounds by categories of foods and specific food items.

## Methods

The NBDPS is one of the largest population-based, case-control studies conducted on causes of birth defects [30]. Beginning in 1998, interviews have been conducted with participants with births affected by selected congenital malformations and comparison (control) participants of unaffected live births identified by surveillance systems in Arkansas, California, Georgia (metropolitan Atlanta), Iowa, Massachusetts, New Jersey, New York, North Carolina, Texas, and Utah. Participants are interviewed by telephone using standardized sets of questions and the Willet Food Frequency Questionnaire (WFFQ) as adapted for this large case-control study [31,32]. This questionnaire collected frequency of intake for 58 food items during the year before pregnancy. Information about maternal health, pregnancy history, diet, substance abuse, occupation, residence, demographics and water use was collected after oral consent was obtained [30]. Completion of the interview occurred between 6 weeks and 2 years after the estimated date of delivery (EDD). For this study, we used data collected from control mothers for births with EDDs from October 1, 1997 through December 31, 2004 and included in version 6.06 of the NBDPS Data.

The WFFQ and several additional questions regarding ethnic foods were used to characterize a mother's dietary intake. The source of information regarding food composition (other than nitrates, nitrites, and nitrosamines) for each item on the questionnaire was the United States Department of Agriculture (USDA) National Nutrient Database for Standard Reference, Release 19 and the WFFQ [31-33]. Daily intake of each food item was calculated based on the frequency of consumption and the average serving size as determined by the WFFQ as adapted for the NBDPS. Using estimates of nitrates, nitrites, and nitrosamines that we developed for the NBDPS-adapted WFFQ [34], daily intake of these compounds was determined for each mother in our study from the frequency of consumption reported for each food item.

### Estimates of nitrates, nitrites, and nitrosamines

We estimated nitrates, nitrites, and nitrosamines for each food item through a multistep process described previously [34]. Briefly, the database was developed by searching the literature for published articles and scientific reports presenting information about the nitrate, nitrite, and/or nitrosamine content in food items. The values were compiled and ranked with respect to time and country of origin. In general, information from 1980 or later and from countries with predominantly western diets were given highest priority and used to generate summary estimates of nitrate, nitrite, or nitrosamine content by food item. The nitrate value for a particular food item was calculated as nitrates in mg/100 g for each food item multiplied by g/serving size. To calculate mg/day of nitrates for each mother, it was necessary to multiply each food item's nitrate serving value (mg/serving) by the number of servings per month and total all applicable food items' contribution to nitrate; the total was divided by 30 days per month, to get the average daily nitrate intake. The process was repeated for nitrites and for nitrosamines. Nitrosamine content from the literature was frequently presented in terms of *N*-nitrosodmethylamine (NDMA), but was also presented as total nitrosamines, specifically identified as *N*-nitrosopyrrolidine (NPYR), *N*-nitrosopiperidine (NPIP), *N*-nitrosoproline (NPOR), or as a combination of the aforementioned. For the purposes of estimating nitrosamine content, total nitrosamines were used either as reported or by adding the values from each of the specific types of nitrosamine--essentially summing the individual values from the reported literature. Estimates for nitrosamines also included contributions from the types and average amount of alcohol intake reported for the first trimester of pregnancy. In the NBDPS, women were asked about the quantity and types of alcohol consumed during pregnancy including the first three months after conception. Total nitrite average daily values were calculated as the average total daily nitrite value + 5% of the average total daily nitrate value. This process produced four dependent variables: average daily intake of dietary nitrate (mg/day), dietary nitrite (mg/day), total dietary nitrite (dietary nitrite + 5% of dietary nitrate, mg/day), and dietary nitrosamine (μg/day).

### Classification of Maternal Characteristics

To identify factors associated with the higher intakes of nitrates, nitrites, and nitrosamines, we developed models using variables from a pre-defined set of factors known to be associated with birth defects. The factors that we considered as covariates were: race/ethnicity (non-Hispanic white, non-Hispanic black, Hispanic, Asian/Pacific Islander, and other); maternal age at conception in years (<20, 20-24, 25-29, 30-34, 35+); maternal education in years at school (0-8, 9-11, 12, 13-15, 16+); annual household income in thousands of dollars (<10, 10-19.999, 20-20.999, 30-30.999, 40-40.999, 50+); intake of folic acid containing supplements (as a single or in a multivitamin supplement, any use one month prior to three months post conception vs. no use); general or multivitamin supplementation (supplements containing more than one vitamin, any use one month prior to three months post conception vs. no use); pre-pregnancy body mass index (BMI) (<18.5 kg/m^2 ^underweight, 18.5-24.9 kg/m^2 ^normal weight, 25.0-29.9 kg/m^2 ^overweight, ≥30.0 kg/m^2 ^obese); area of residence (Arkansas, California, Georgia, Iowa, Massachusetts, New Jersey, New York, North Carolina, Texas, and Utah); dietary folate intake as dietary folate equivalents in quartiles (DFE) (<319; 319-464.9; 465-685.5; >685.5 μg/day); and dietary fat (percentage of daily caloric intake ≤30%, >30%). Additionally, information was available on frequency and type of alcohol consumed (beer, wine, malt liquor, mixed drinks, and shot liquor). Nitrosamine values for alcohol were estimated from the available literature for nitrosamine content only. Very few sources were located that reported the nitrate and nitrite content of alcoholic beverages, therefore estimated intake of nitrates and nitrites from alcohol was not calculated.

### Statistical Analysis

Multinomial logistic regression models (STATA 10) were used to calculate odds ratios and 95% confidence intervals of the relation between various maternal characteristics and quartile of intake of nitrates, nitrites, total nitrites (nitrites + 5% nitrates), and nitrosamines [35]. In addition to examining each factor individually to identify inconsistencies or errors, Pearson correlations between predictor variables were also examined using STATA 10 to identify any potential collinearity problems [35]. Based on a predefined correlation coefficient of ≥ 0.80 as the threshold for further investigation, we found no variables to exclude. The bivariate analyses of each potential predictor variable (maternal characteristic) with each dependent variable (dietary nitrate intake, dietary nitrite intake, total dietary nitrite intake, and dietary nitrosamine intake) were examined. The significance of the association with dietary intake of nitrates, nitrites, total nitrites, and nitrosamines was assessed for each potential predictor variable using the likelihood ratio chi-square test.

Using a backward elimination method we began each model with all potential predictor variables that were not excluded based on likelihood ratio test of significance for association with the dependent variable (p-value 0.20). With the exception of race/ethnicity (our main predictor variable of interest), each covariate was removed sequentially and the Bayesian Information Criteria (BIC) recorded for each specific model. If an odds ratio for any race/ethnicity estimate at any quartile of intake changed by 10% or more, the variable remained in the model as a potential confounder. Interactions between race/ethnicity and education, race/ethnicity and age, and race/ethnicity and location were also examined. Likelihood ratio tests were used to evaluate models with the interaction terms compared with models without the interaction terms and reviewed for trends. The numbers of participants were too few based on our categorization of the variables to accurately determine significance of interaction terms as specified in our models.

## Results

A total of 5,958 control participants were included in the study with estimated dates of delivery during the period 1997-2004. Approximately 68% of eligible controls participated in the interview, and the average interval between the estimated date of delivery and these interviews was about 8 months.

Although information from the WFFQ as adapted for the NBDPS was available for all participants (n = 5,958), approximately 97% had complete dietary information for calculation of dietary nitrate (n = 5,809), nitrite (n = 5,818), total nitrite (n = 5,809), and nitrosamine intake (n = 5,803). Quartiles of each dependent variable were: nitrate (≤27.059; 27.060 - 40.485; 40.486 - 60.602; and >60.602 mg/day; n = 5,809); nitrite (≤1.12224;1.12225 - 1.53386; 1.53387 - 2.13050; >2.13050 mg/day; n = 5,818); total nitrite (≤ 2.63578; 2.63579 - 3.69108; 3.69109 - 5.21853; >5.21853 mg/day; n = 5,809); nitrosamines (≤0.33299; 0.33300 - 0.47158; 0.47159 - 0.66846; >0.66846 μg/day; n = 5,837).

We summarize the associations found between maternal characteristics and quartiles of dietary intake for nitrates, nitrites, total nitrites, and nitrosamines in Tables 1, 2, 3, and 4 respectively and also present the crude and adjusted odds ratios for significant variables included in the model. These tables show that maternal race/ethnicity, age, education, household income, folic acid supplementation, area of residence, dietary folate intake, and dietary fat consumption were each significantly (p-value < 0.05) associated with nitrate, nitrite, total nitrite, and nitrosamine intake. General or multi-vitamin use was significantly associated with intake of nitrites and nitrosamines, and pre-pregnancy BMI was significantly associated with nitrite intake. In general, participants who consumed nitrate, nitrite, and total dietary nitrite at the lowest quartile of intake were non-Hispanic White, younger, more educated, consumed less dietary fat, and had greater household income than participants at the highest quartile of intake. In contrast, participants who consumed nitrosamines at the lowest quartile of intake were non-Hispanic Black, Hispanic, or Asian/Pacific Islander, less than 20 years of age at conception, less educated, normal or underweight, and consumed less fat than participants at the highest quartile of nitrosamine intake.

**Table 1:** Maternal characteristics associated with dietary nitrate intake, National Birth Defects Prevention Study Controls, 1997-2004

		Dietary nitrate intake (mg/day)									**Odds ratios and 95% confidence intervals**^**b**^	
				
		**Quartile 1**^**a**^		Quartile 2		Quartile 3		Quartile 4				
	
		<27.06		27.07-40.48		40.49-60.60		61.89-809.64		p-value	4th quartile compared to the 1st quartile	
	
		n	%	n	%	n	%	n	%		Crude	**Adjusted**^**c**^
	
Race/Ethnicity												
White non-Hispanic		991	28.4	964	27.6	890	25.5	647	18.5	<0.0001	1.0	1.0
Black non-Hispanic		130	19.7	119	18.1	167	25.3	243	36.9		2.9 (2.3,3.6)	2.4 (1.7,3.3)
Hispanic		267	20.6	292	22.6	318	24.6	417	32.2		2.4 (2.0,2.9)	1.4 (1.0,1.9)
Asian/Pacific Islander		25	14.9	19	11.3	36	21.4	88	52.4		5.4 (3.4,8.5)	4.8 (2.7,8.5)
Other		34	19.4	51	29.2	39	22.3	51	29.1		2.3 (1.5,3.6)	1.6 (0.89,2.9)
Missing		6	28.6	7	33.3	2	9.5	6	28.6		---	---
**State/Area of residence**												
Texas		168	**24.5**	192	**27.9**	165	**24.0**	162	**23.6**	<0.0001	1.0	1.0
Arkansas		194	**27.5**	182	**25.9**	168	**23.8**	161	**22.8**		0.86 (0.64,1.2)	0.79 (0.52,1.2)
California		191	**24.5**	165	**21.2**	197	**25.3**	226	**29.0**		1.2 (0.92,1.6)	0.98 (0.68,1.4)
Georgia		129	**21.1**	127	**20.8**	165	**27.1**	189	**31.0**		**1.5 (1.1,2.1)**	**2.2 (1.4,3.4)**
Iowa		245	**37.9**	187	**28.9**	147	**22.8**	67	**10.4**		**0.30 (0.20,0.40)**	0.37 (0.23,1.4)
Massachusetts		150	**20.2**	192	**25.9**	216	**29.1**	184	**24.8**		1.3 (0.94,1.7)	**2.9 (1.9,4.5)**
New Jersey		103	**18.2**	122	**21.5**	152	**26.9**	189	**33.4**		**1.9 (1.4,2.6)**	**2.7 (1.7,4.1)**
New York		127	**24.2**	126	**24.0**	135	**25.8**	136	**26.0**		1.1 (0.80,1.5)	**1.8 (1.1,2.8)**
North Carolina		72	**24.7**	81	**27.7**	54	**18.5**	85	**29.1**		1.2 (0.84,1.8)	**1.7 (1.1,2.9)**
Utah		74	**28.7**	78	**30.3**	53	**20.5**	53	**20.5**		0.74 (0.49,1.1)	0.99 (0.59,1.7)

**Dietary folate equivalent (μg/day)**												
<319		725	**50.4**	422	**29.3**	202	**14.1**	89	**6.2**	<0.0001	1.0	1.0
319-464.9		351	**24.0**	463	**31.6**	416	**28.5**	233	**15.9**		**5.4 (4.1,7.1)**	**3.4 (2.5,4.7)**
465-685.5		232	**15.9**	350	**23.9**	451	**30.9**	429	**29.3**		**15.1 (11.5,19.8)**	**5.5 (4.0,7.6)**
> 685.5		145	**10.0**	217	**15.0**	383	**26.5**	701	**48.5**		**39.4 (29.7,52.3)**	**7.3 (5.1,10.3)**

**Maternal household income (dollars annually)**												
≥50 K		442	**24.1**	469	**25.6**	496	**27.0**	428	**23.3**	<0.0001	1.0	1.0
40-49,999		120	**28.4**	114	**27.0**	100	**23.6**	89	**21.0**		0.77 (0.56,1.0)	**0.67 (0.47,0.97)**
30-39,999		138	**25.5**	153	**28.3**	134	**24.8**	116	**21.4**		0.87 (0.66,1.1)	0.71 (0.50,1.0)
20-29,999		180	**24.2**	203	**27.2**	182	**24.4**	180	**24.2**		1.0 (0.81,1.3)	0.71 (0.52,0.97)
10-19.999		165	**22.9**	167	**23.1**	189	**26.2**	201	**27.8**		1.3 (0.98,1.6)	**0.49 (0.36,0.68)**
< 10,000		233	**25.2**	205	**22.1**	198	**21.4**	290	**31.3**		1.3 (1.0,1.6)	**0.43 (0.32,0.59)**
Missing		175	**28.4**	141	**22.9**	153	**24.8**	148	**24.0**		---	---

**Maternal education (years at school)**												
16+		436	**23.8**	465	**25.5**	483	**26.4**	445	**24.3**	0.0010	1.0	---
13-15		399	**25.9**	393	**25.5**	377	**24.5**	371	**24.1**		0.91 (0.75,1.1)	---
12		394	**27.4**	361	**25.1**	338	**23.5**	345	**24.0**		0.86 (0.71,1.0)	---
9-11		171	**15.7**	166	**15.2**	361	**33.1**	393	**36.0**		1.1 (0.82,1.3)	---
0-8		46	**16.7**	60	**21.7**	71	**25.7**	99	**35.9**		2.1 (1.5,3.1)	---
Missing		7	**24.1**	7	**24.1**	7	**24.1**	8	**27.7**		---	---

**Dietary Fat (% of daily calories)**												
≤ 30%		768	**23.8**	762	**23.5**	828	**25.7**	870	**27.0**	<0.0001	1.0	---
>30%		685	**26.6**	690	**26.7**	624	**24.2**	582	**22.5**		0.75 (0.65,0.87)	---

**Pre-pregnancy BMI**												
Normal weight (18.5 < 25.0)		67	**21.3**	81	**25.8**	82	**26.1**	84	**26.8**	0.6610	1.0	---
Under weight (<18.5)		813	**25.9**	775	**24.7**	783	**25.0**	765	**24.4**		1.3 (0.55,1.9)	---
Over weight (25.0 < 30.0)		315	**25.3**	305	**24.5**	325	**26.1**	299	**24.0**		1.0 (0.84,1.2)	---
Obese (≥30.0)		215	**24.2**	241	**27.2**	212	**23.9**	219	**24.7**		1.1 (0.88,1.3)	---
Missing		43	**18.9**	50	**21.9**	50	**21.9**	85	**37.3**		---	---

**Folic acid containing supplements**												
No		687	**26.4**	617	**23.7**	617	**23.7**	678	**26.2**	0.0070	1.0	---
Yes		758	**23.9**	828	**26.0**	827	**26.0**	765	**24.1**		1.0 (0.88,1.2)	---
Missing		8	**25.0**	7	**21.9**	8	**25.0**	9	**28.1**		---	---

**Multivitamin use**												
No		876	**24.6**	905	**25.4**	898	**25.2**	885	**24.8**	0.7780	1.0	---
Yes		568	**25.6**	546	**24.6**	548	**24.7**	558	**25.1**		0.97 (0.84,1.3)	---
Missing		9	**36.0**	1	**4.0**	6	**24.0**	9	**36.0**		---	---

**Maternal age at conception (years)**												
20-24		409	**30.5**	328	**24.5**	306	**22.9**	296	**22.1**	<0.0001	1.0	---
<20		241	**29.3**	213	**25.8**	189	**23.0**	180	**21.9**		1.0 (0.80,1.3)	---
25-29		371	**23.8**	410	**26.3**	382	**24.5**	397	**25.4**		1.5 (1.2,1.8)	---
30-34		304	**21.3**	364	**25.6**	390	**27.3**	368	**25.8**		1.7 (1.4,2.1)	---
35+		128	**19.4**	137	**20.7**	185	**28.0**	211	**31.9**		2.3 (1.8,2.9)	---

^a ^Referent category is nitrate intake at the lowest quartile of intake (27.06 mg.day)

^b ^Only presented for variables included in the final multinomial logistic regression model

^c ^Adjusted for tertiles of daily caloric intake, race/ethnicity, state of residence, maternal household income, and dietary folate as dietary folate equivalents.

Note: p-value excludes missing values

Note: missing categories contain refuse, don't know, and missing

**Table 2:** Maternal characteristics associated with dietary nitrite intake, National Birth Defects Prevention Study Controls, 1997-2004

		Nitrite intake (mg/day)									**Odds ratios and 95% confidence intervals**^**b**^	
			
		**Quartile 1**^**a**^		Quartile 2		Quartile 3		Quartile 4				
		
		0.10123 - 1.12224		1.12225 - 1.53386		1.53386 - 2.1305		>2.1305		p-value	4th quartile compared to the 1st quartile	
	
		n	%	n	%	n	%	n	%		Crude	**Adjusted**^**b**^
										
Race/Ethnicity												
White non-Hispanic		1044	29.9	1016	29.1	889	25.4	548	15.7	<0.0001	1.0	1.0
Black non-Hispanic		155	23.5	138	20.9	182	27.6	185	28.0		2.3 (1.8,2.9)	1.9 (1.3,2.7)
Hispanic		166	12.8	214	16.5	305	23.6	610	47.1		7.0 (5.7,8.6)	6.2 (4.3,9.0)
Asian/Pacific Islander		33	19.5	42	24.9	36	21.3	58	34.3		3.3 (2.2,5.2)	10.3 (5.4,19.6)
Other		45	25.6	40	22.9	40	22.9	50	28.6		2.1 (1.4,3.2)	2.2 (1.2,4.2)
Missing		12	54.5	4	18.3	3	13.6	3	13.6		---	---
**State/Area of residence**												
Texas		106	**15.4**	128	**18.7**	171	**24.9**	282	**41.0**	<0.0001	1.0	1.0
Arkansas		145	**20.5**	152	**21.5**	196	**27.6**	215	**30.4**		**0.56 (0.41,0.76)**	0.88 (0.55,1.4)
California		149	**19.1**	145	**18.5**	199	**25.4**	289	**37.0**		**0.73 (0.54,0.98)**	**0.49 (0.32,0.76)**
Georgia		165	**27.0**	155	**25.3**	153	**25.0**	139	**22.7**		**0.28 (0.20,0.38)**	0.67 (0.42,1.1)
Iowa		184	**28.5**	155	**24.0**	171	**26.4**	136	**21.1**		**0.11 (0.08,0.16)**	**0.30 (0.18,0.50)**
Massachusetts		234	**31.5**	264	**35.6**	175	**23.6**	69	**9.3**		**0.31 (0.23,0.43)**	0.50 (0.30,0.82)
New Jersey		156	**27.6**	145	**25.6**	135	**23.8**	130	**23.0**		**0.22 (0.16,0.3)**	**0.51 (0.31,0.86)**
New York		161	**30.7**	143	**27.2**	125	**23.8**	96	**18.3**		**0.32 (0.23,0.43)**	**0.54 (0.33,0.89)**
North Carolina		74	**25.3**	87	**29.8**	73	**25.0**	58	**19.9**		**0.29 (0.20,0.44)**	**0.45 (0.24,0.82)**
Utah		81	**31.4**	80	**31.0**	57	**22.1**	40	**15.5**		**0.19 (0.12,0.29)**	**0.22 (0.12,0.41)**

**Dietary folate equivalent (μg/day)**												
<319		670	**27.7**	398	**27.6**	267	**18.5**	105	**7.3**	<0.0001	1.0	1.0
319-464.9		369	**25.2**	440	**30.0**	410	**28.0**	246	**16.8**		**4.3 (3.3,5.5)**	**1.9 (1.3,2.6)**
465 - 685.5		267	**18.2**	374	**25.5**	430	**29.4**	394	**26.9**		**9.4 (7.3,12.2)**	**1.8 (1.3,2.6)**
> 685.5		149	**10.3**	242	**16.7**	348	**24.0**	709	**49.0**		**30.4 (23.2,39.8)**	**2.3 (1.5,3.3)**

**Maternal household income (dollars annually)**												
≥50,000		559	**30.5**	582	**31.7**	452	**24.6**	243	**13.2**	<0.0001	1.0	1.0
40-49,999		117	**27.7**	119	**28.1**	106	**25.1**	81	**19.1**		**1.6 (1.2,2.2)**	1.0 (0.66,1.6)
30-39,999		138	**25.5**	130	**24.0**	143	**26.4**	131	**24.1**		**2.2 (1.6,2.9)**	1.4 (0.92,2.0)
20-29,999		168	**22.6**	178	**23.9**	188	**25.2**	211	**28.3**		**2.9 (2.2,3.7)**	1.2 (0.81,1.7)
10-19.999		132	**18.3**	133	**18.4**	192	**26.5**	266	**36.8**		**4.6 (3.6,6.0)**	1.0 (0.68,1.5)
< 10,000		196	**21.1**	166	**17.8**	201	**21.6**	368	**39.5**		**4.3 (3.4,5.4)**	0.71 (0.48,1.1)
Missing		145	**23.5**	146	**23.6**	173	**28.0**	154	**24.9**		---	---

**Maternal education (years at school)**												
16+		538	**29.4**	585	**32.0**	456	**24.9**	251	**13.7**	<0.0001	1.0	1.0
13-15		374	**24.3**	388	**25.2**	428	**27.8**	352	**22.7**		**2.0 (1.6,2.5)**	1.3 (0.98,1.8)
12		356	**24.7**	316	**21.9**	332	**23.0**	438	**30.4**		**2.6 (2.1,3.2)**	1.2 (0.88,1.7)
9-11		145	**20.8**	112	**16.1**	171	**24.5**	269	**38.6**		**4.0 (3.1,5.1)**	1.2 (0.79,1.9)
0-8		31	**11.2**	47	**17.0**	63	**22.7**	136	**49.1**		**9.4 (6.2,14.3)**	**2.9 (1.4,5.8)**
Missing		11	**36.7**	6	**20.0**	5	**16.7**	8	**26.7**		---	---

**Dietary Fat (% of daily calories)**												
≤ 30%		950	**29.4**	827	**25.6**	756	**23.4**	702	**21.6**	<0.0001	1.0	1.0
>30%		505	**19.6**	627	**24.2**	699	**27.1**	752	**29.1**		**2.0 (1.7,2.3)**	**8.7 (6.9,11.0)**

**Pre-pregnancy BMI**												
Normal weight (18.5 < 25.0)		882	**28.1**	820	**26.1**	734	**23.4**	705	**22.4**	<0.0001	1.0	---
Under weight (<18.5)		80	**25.4**	74	**23.5**	80	**25.4**	81	**25.7**		1.3 (0.92,1.8)	---
Over weight (25.0 < 30.0)		286	**23.0**	320	**25.7**	332	**26.7**	307	**24.6**		1.3 (1.1,1.6)	---
Obese (≥30.0)		184	**20.7**	198	**22.3**	257	**28.9**	249	**28.1**		1.7 (1.4,2.1)	---
Missing		23	**10.0**	42	**18.3**	52	**22.8**	112	**48.9**		---	---

**Folic acid containing supplements**												
No		600	**23.1**	558	**21.4**	646	**24.8**	799	**30.7**	<0.0001	1.0	---
Yes		846	**26.6**	888	**27.9**	799	**25.1**	650	**20.4**		0.57 (0.50,0.67)	---
Missing		9	**28.1**	8	**25.0**	10	**31.3**	5	**15.6**		---	---

**Multivitamin use**												
No		891	**25.0**	951	**26.6**	898	**25.2**	829	**23.2**	<0.0001	1.0	---
Yes		556	**25.0**	502	**22.6**	552	**24.8**	613	**27.6**		1.2 (1.0,1.4)	---
Missing		8	**30.8**	1	**3.8**	5	**19.2**	12	**46.2**		---	---

**Age at conception (years)**												
20-24		325	**24.2**	307	**22.9**	323	**24.1**	386	**28.8**	<0.0001	1.0	
<20		204	**24.7**	165	**20.0**	185	**22.3**	273	**33.0**		1.1 (0.89,1.4)	---
25-29		379	**24.3**	409	**26.2**	391	**25.0**	382	**24.5**		0.85 (0.69,1.0)	---
30-34		372	**26.1**	383	**26.8**	382	**26.7**	291	**20.4**		0.66 (0.53,0.81)	---
35+		175	**26.5**	190	**28.7**	174	**26.3**	122	**18.5**		0.59 (0.44,0.77)	---

^a^Referent category is nitrite intake at the lowest quartile of intake (<1.2225 mg.day)

^b^Only presented for variables included in the final multinomial logistic regression model

^c^Adjusted for tertiles of daily caloric intake, race/ethnicity, state of residence, maternal education, dietary fat intake, maternal household income, and dietary folate as dietary folate equivalents

Note: p-value excludes missing values

Note: missing categories contain refuse, don't know, and missing

**Table 3:** Maternal characteristics associated with total dietary nitritea intake, National Birth Defects Prevention Study Controls, 1997-2004

		Total Nitrite intake (mg/day)									**Odds ratios and 95% confidence intervals**^**c**^	
			
		**Quartile 1**^**b**^		Quartile 2		Quartile 3		Quartile 4				
		
		0.31362-2.63578		2.63579 - 3.69108		3.69109 - 5.21853		5.21854 - 59.0717		p-value	4th quartile compared to the 1st quartile	
	
		n	%	n	%	n	%	n	%		Crude	**Adjusted**^**d**^
										
Race/Ethnicity												
White non-Hispanic		1024	29.3	988	28.3	903	25.9	577	16.5	<0.0001	1.0	1.0
Black non-Hispanic		132	20	134	20.4	151	22.9	242	36.7		3.3 (2.6,4.1)	2.6 (1.8,3.6)
Hispanic		222	17.2	263	20.3	315	24.3	494	38.2		3.9 (3.3,4.8)	2.0 (1.5,2.8)
Asian/Pacific Islander		28	16.7	19	11.3	39	23.2	82	48.8		5.2 (3.3, 8.1)	9.2 (5.1,16.7)
Other		39	22.3	42	24	43	24.6	51	29.1		2.3 (1.5,3.6)	1.9 (1.1,3.5)
Missing		8	38.1	6	28.6	1	4.7	6	28.6		---	---
**State/Area of residence**												
Texas		140	**20.4**	171	**24.8**	166	**24.2**	210	**30.6**	<0.0001	1.0	1.0
Arkansas		178	**25.2**	181	**25.7**	163	**23.1**	183	**26**		**0.69 (0.51,0.93)**	0.76 (0.50,1.2)
California		176	**22.6**	162	**20.8**	188	**24.1**	253	**32.5**		0.96 (0.72,1.3)	0.84 (0.57,1.2)
Georgia		137	**22.5**	135	**22.1**	159	**26.1**	179	**29.3**		**0.24 (0.18,0.34)**	0.40 (0.25,0.63)
Iowa		229	**35.4**	178	**27.6**	155	**24.0**	84	**13**		**0.54 (0.40,0.74)**	**1.7 (1.1,2.7)**
Massachusetts		173	**23.3**	213	**28.7**	215	**29.0**	141	**19**		0.97 (0.70,1.3)	**1.7 (1.1,2.6)**
New Jersey		114	**20.1**	141	**24.9**	145	**25.7**	166	**29.3**		**0.51 (0.37,0.71)**	1.0 (0.65,1.6)
New York		147	**28.1**	125	**23.8**	139	**26.5**	113	**21.6**		0.87 (0.64,1.2)	1.5 (0.93,2.3)
North Carolina		76	**26**	73	**25**	66	**22.6**	77	**26.4**		**0.68 (0.46,0.99)**	1.3 (0.76,2.2)
Utah		83	**32.2**	73	**28.3**	56	**21.7**	46	**17.8**		**0.37 (0.24,0.56)**	**0.51 (0.29,0.90)**

**Dietary folate equivalent (μg/day)**												
<319		739	**51.4**	427	**29.6**	208	**14.5**	64	**4.5**	<0.0001	1.0	1.0
319-464.9		349	**23.9**	463	**31.6**	441	**30.1**	210	**14.4**		**6.9 (5.1,9.4)**	**4.0 (2.9,5.7)**
465-685.5		232	**15.9**	352	**24.1**	452	**30.9**	426	**29.1**		**21.2 (15.7,28.7)**	**6.4 (4.5,9.1)**
>685.5		133	**9.2**	210	**14.5**	351	**24.3**	752	**52**		**65.3 (47.6,89.5)**	**8.7 (6.0,12.6)**

**Folic acid containing supplements**												
No		646	**24.9**	606	**23.3**	625	**24.0**	722	**27.8**	<0.0001	1.0	1.0
Yes		799	**25.1**	837	**26.3**	820	**25.8**	722	**22.7**		0.81 (0.70,0.94)	1.1 (0.89,1.3)
Missing		8	**25**	9	**28.1**	7	**21.9**	8	**25**		---	---

**Maternal education (years at school)**												
16+		473	**25.9**	517	**28.3**	458	**25.0**	381	**20.8**	<0.0001	1.0	1.0
12-15		390	**25.3**	393	**25.5**	400	**26.0**	357	**23.2**		1.1 (093,1.4)	0.80 (0.61,1.0)
12		377	**26.2**	346	**24.1**	349	**24.3**	366	**25.5**		1.2 (0.99,1.5)	**0.55 (0.42,0.73)**
9-11		164	**23.5**	142	**20.4**	163	**23.4**	228	**32.7**		**1.7 (1.4,2.2)**	**0.49 (0.34,0.70)**
0-8		42	**15.2**	46	**16.7**	74	**26.8**	114	**41.3**		**3.4 (2.3,4.9)**	0.87 (0.46,1.7)
Missing		7	**24.1**	8	**27.6**	8	**27.6**	6	**20.7**		---	---

**Dietary Fat (% of daily calories)**												
< 30%		844	**26.1**	773	**23.9**	786	**24.4**	825	**25.6**	0.0280	1.0	1.0
>30%		609	**23.6**	679	**26.3**	666	**25.8**	627	**24.3**		**1.1 (0.91,1.2)**	**2.5 (2.0,3.0)**

**Pre-pregnancy BMI**												
Normal weight (18.5 < 25.0)		843	**26.9**	772	**24.6**	772	**24.6**	749	**23.9**	0.1870	1.0	1.0
Under weight (<18.5)		72	**22.9**	80	**25.5**	78	**24.8**	84	**26.8**		1.3 (0.94,1.8)	1.1 (0.73,1.7)
Over weight (25.0 < 30.0)		301	**24.2**	327	**26.2**	323	**26.0**	293	**23.6**		1.1 (0.91,1.3)	1.3 (0.80,2.0)
Obese (>30.0)		203	**22.9**	231	**26**	213	**24.0**	240	**27.1**		**1.3 (1.1,1.6)**	**1.8 (1.1,2.9)**
Missing		34	**14.9**	42	**18.5**	66	**28.9**	86	**37.7**		---	---

**Maternal household income (dollars annually)**												
≥50,000		484	**26.4**	516	**28.1**	474	**25.8**	361	**19.7**	<0.0001	1.0	---
40-49,999		116	**27.4**	122	**28.8**	94	**22.3**	91	**21.5**		1.1 (0.77,1.4)	---
30-39,999		150	**27.7**	125	**23.1**	148	**27.4**	118	**21.8**		1.1 (0.80,1.4)	---
20-29,999		176	**23.6**	184	**24.7**	204	**27.4**	181	**24.3**		1.4 (1.1,1.8)	---
10-19.999		157	**21.7**	150	**20.9**	180	**24.9**	235	**32.5**		2.0 (1.6,2.6)	---
< 10,000		211	**22.8**	189	**20.4**	207	**22.4**	319	**34.4**		2.0 (1.6,2.5)	---
Missing		159	**25.8**	166	**26.9**	145	**23.5**	147	**23.8**		---	---

**Multivitamin use**												
No		882	**24.7**	914	**25.7**	905	**25.4**	863	**24.2**	0.2470	1.0	---
Yes		563	**25.4**	537	**24.2**	539	**24.2**	581	**26.2**		1.1 (0.91,12)	---
Missing		8	**32**	1	**4**	8	**32.0**	8	**32**		---	---

**Age at conception**												
20-24		377	**28.2**	320	**23.9**	314	**23.4**	328	**24.5**	0.002	1.0	
<20		230	**27.9**	200	**24.4**	188	**22.8**	205	**24.9**		1.0 (0.81,1.3)	---
25-29		389	**24.9**	385	**24.7**	386	**24.8**	400	**25.6**		1.2 (0.96,1.4)	---
30-34		317	**22.2**	375	**26.3**	400	**28.1**	334	**23.4**		1.2 (0.98,1.5)	---
35+		140	**21.2**	172	**26**	164	**24.8**	185	**28**		1.5 (1.2,1.9)	---

^a^Total dietary nitrite = 5% dietary nitrate + dietary nitrite

^b^Referent category is total nitrite intake at the lowest quartile of intake (<2.63578 mg/day)

^c^Only presented for variables included in the final multinomial logistic regression model

^d^Adjusted for tertiles of daily caloric intake, race/ethnicity, maternal education, state of residence, dietary fat intake, pre-pregnancy body mass index, and folic acid supplementation

Note: p-value excludes missing values

Note: missing categories contain refuse, don't know, and missing

**Table 4:** Maternal characteristics associated with dietary nitrosamine intake, National Birth Defects Prevention Study Controls, 1997-2004

		Nitrosamine intake μg/day									**Odds ratios and 95% confidence intervals**^**b**^	
			
		**Quartile 1**^**a**^		Quartile 2		Quartile 3		Quartile 4				
		
		<0.3330		0.3330-0.4715		0.4716-0.6685		>0.6685		p-value	4th quartile compared to the 1st quartile	
	
		n	%	n	%	n	%	n	%		Crude	**Adjusted**^**c**^
			
Race/Ethnicity												
White non-Hispanic		761	21.8	894	25.7	917	26.3	912	26.2	<0.0001	1.0	1.0
Black non-Hispanic		203	30.8	158	23.9	152	23.0	147	22.3		0.60 (0.48,0.76)	0.36 (0.26,0.50)
Hispanic		357	27.5	315	24.2	318	24.5	310	23.8		0.73 (0.61,0.87)	0.35 (0.26,0.48)
Asian/Pacific Islander		68	40.2	32	18.9	29	17.2	40	23.7		0.49 (0.33,0.73)	0.33 (0.19,0.57)
Other		53	31.6	45	26.8	34	20.2	36	21.4		0.57 (0.37,0.87)	0.42 (0.24,0.74)
Missing		9	40.9	7	31.8	1	4.5	5	22.7			
**Area of residence (state)**												
Texas		168	**24.6**	166	**24.3**	176	**25.7**	174	**25.4**	<0.0001	1.0	1.0
Arkansas		151	**21.4**	150	**21.2**	193	**27.3**	213	**30.1**		1.4 (1.0,1.8)	1.0 (0.69,1.6)
California		235	**30.1**	174	**22.2**	171	**21.9**	202	**25.8**		0.83 (0.63,1.1)	0.73 (0.51,1.0)
Georgia		185	**30.1**	158	**25.7**	151	**24.7**	120	**19.5**		**2.5 (1.8,3.4)**	**3.6 (2.4,5.5)**
Iowa		100	**15.5**	118	**18.3**	168	**26.1**	258	**40.1**		**0.55 (0.40,0.75)**	0.66 (0.44,1.0)
Massachusetts		208	**28.0**	226	**30.4**	191	**25.7**	118	**15.9**		0.84 (0.61,1.2)	0.87 (0.57,1.3)
New Jersey		141	**25.0**	160	**28.4**	141	**25.0**	122	21.6		**0.75 (0.54,1.0)**	0.82 (0.53,1.3)
New York		135	26.0	156	30.1	123	23.7	105	20.2		**0.63 (0.46,0.86)**	0.67 (0.44,1.0)
North Carolina		84	**29.0**	87	**30.0**	64	**22.0**	55	**19.0**		**0.63 (0.42,0.94)**	**0.49 (0.29,0.83)**
Utah		44	**17.2**	56	**21.9**	73	**28.5**	83	**32.4**		**1.8 (1.2,2.8)**	1.8 (1.0,3.0)

**Dietary folate (μg/day)**												
<319		619	**43.0**	386	**26.8**	273	**19.1**	160	**11.1**	<0.0001	1.0	1.0
319-465		362	**24.9**	414	**28.5**	395	**27.2**	281	**19.4**		**3.0 (2.4,3.8)**	**1.7 (1.3,2.3)**
465 - ≤685.5		288	**19.7**	368	**25.2**	416	**28.4**	390	**26.7**		**5.2 (4.2,6.6)**	**2.0 (1.5,2.7)**
> 685.5		182	**12.5**	283	**19.5**	367	**25.3**	619	**42.7**		**13.2 (10.4,16.7)**	**3.4 (2.4,4.7)**

**Maternal education (years at school)**												
16+		402	**22.0**	508	**27.8**	485	**26.6**	431	**23.6**	<0.0001	1.0	1.0
12-15		343	**22.3**	384	**24.9**	404	**26.2**	409	**26.6**		1.1 (0.91,1.4)	**0.74 (0.58,0.96)**
12		404	**28.0**	356	**24.7**	330	**22.8**	354	**24.5**		**0.82 (0.67,0.99)**	**0.38 (0.29,0.50)**
9-11		209	**30.0**	143	**20.6**	161	**23.1**	183	**26.3**		0.82 (0.64,1.0)	**0.33 (0.24,0.47)**
0-8		83	**29.9**	57	**20.5**	67	**24.1**	71	**25.5**		0.80 (0.57,1.1)	**0.37 (0.23,0.61)**
Missing		10	**52.6**	3	**15.8**	4	**21.1**	2	**10.5**			

**Dietary fat (% of daily calories)**												
≤ 30%		1034	**31.9**	871	**26.9**	726	**22.5**	606	**18.7**	<0.0001	1.0	1.0
>30%		417	**16.3**	580	**22.5**	725	**28.3**	844	**32.9**		**3.5 (3.0,4.0)**	**8.5 (6.9,10.4)**

**Maternal household income (dollars annually)**												
≥50,000		415	**22.7**	517	**28.2**	490	**26.8**	409	**22.3**	<0.0001	1.0	---
40-49,999		92	**21.8**	114	**27.0**	91	**21.6**	125	**29.6**		1.4 (1.0,1.9)	---
30-39,999		123	**22.8**	135	**25.0**	141	**26.1**	141	**26.1**		1.2 (0.88,1.5)	---
20-29,999		192	**25.8**	182	**24.4**	196	**26.3**	175	**23.5**		0.92 (0.72,1.2)	---
10-19.999		173	**24.0**	181	**25.0**	165	**22.9**	203	**28.1**		1.2 (0.93,1.5)	---
< 10,000		267	**28.6**	199	**21.4**	223	**23.9**	243	**26.1**		0.92 (0.74,1.2)	---
Missing		189	**30.9**	123	**20.0**	145	**23.7**	154	**25.2**		---	---

**Pre-pregnancy BMI**												
Normal weight (18.5 - 25.0)		792	**25.2**	772	**24.6**	790	**25.2**	783	**25.0**	<0.0001	1.0	---
Under weight (<18.5)		89	**28.3**	86	**27.4**	75	**23.9**	64	**20.4**		0.73 (0.52,1.0)	---
Over weight (25.0 - 30.0)		291	**23.5**	321	**25.8**	317	**25.6**	311	**25.1**		1.1 (0.90,1.31)	---
Obese (>30.0)		206	**23.3**	209	**23.7**	222	**25.1**	246	**27.9**		1.2 (0.97,1.5)	---
Missing		73	**31.9**	63	**27.5**	47	**20.5**	46	**20.1**		---	---

**Folic acid containing supplements**												
No		715	**27.5**	599	**23.0**	624	**24.1**	661	**25.4**	<0.0001	1.0	---
Yes		725	**22.8**	844	**26.6**	824	**25.9**	783	**24.7**		1.2 (1.0,1.4)	---
Missing		11	**39.3**	8	**28.6**	3	**10.7**	6	**21.4**		---	---

**Multivitamin use**												
No		864	**24.3**	935	**26.3**	896	**25.2**	860	**24.2**	0.0020	1.0	---
Yes		579	**26.0**	511	**23.0**	551	**24.8**	582	**26.2**		1.0 (0.87,1.2)	---
Missing		8	**32.0**	5	**20.0**	4	**16.0**	8	**32.0**		---	---

**Age at conception**												
20-24		348	**25.8**	317	**23.5**	331	**24.6**	351	**26.1**	<0.0001	1.0	---
<20		274	**33.4**	171	**20.9**	184	**22.4**	191	**23.3**		0.69 (0.54,0.88)	---
25-29		350	**22.5**	418	**26.9**	391	**25.1**	397	**25.5**		1.1 (0.91,1.4)	---
30-34		330	**23.2**	375	**26.4**	356	**25.1**	359	**25.3**		1.1 (0.87,1.3)	---
35+		149	**22.6**	170	**25.8**	189	**28.6**	152	**23.0**		1.0 (0.77,1.3)	---

^a^Referent category is nitrosamine intake at the lowest quartile of intake (<0.33299 μg/day)

^b^Only presented for variables included in the final multinomial logistic regression model

^c^Adjusted for tertiles of daily caloric intake, race/ethnicity, maternal education, state of residence, dietary fat intake, and dietary folate as dietary folate equivalents. intake, pre-pregnancy body mass index, and folic acid supplementation

Note: p-value excludes missing values

Tables 1, 2, 3, and 4 also respectively display the crude and adjusted odds ratios with 95% confidence intervals for the association of consumption of nitrate, nitrite, total nitrite, and nitrosamine intake in the highest quartile and maternal characteristics. Odds ratios were further adjusted for tertiles of daily caloric intake (<1232.6 kcal, 1232.6-1740.7 kcal, 1740.7 or more kcal). The lowest quartile of intake was used as the referent category for each compound. Additional File 1 contains tables with the results of all quartiles of estimated intake.

### Nitrate intake

Maternal race and ethnicity were important predictors of dietary nitrate intake; participants categorized as Asian/Pacific Islanders were 4.8 times more likely (95% CI 2.7-8.5) than non-Hispanic White participants to consume nitrates at the highest quartile of intake (Table 1). Residence was also an important factor for nitrate consumption. Compared to Texas participants, those in Georgia, Massachusetts, New Jersey, New York, and North Carolina were significantly more likely to consume nitrates at the fourth quartile of intake. Participants who reported household incomes of <$50,000 annually were less likely to consume nitrate at the highest quartile of intake compared to those with household incomes $50,000 or more annually. Participants whose daily dietary folate intake exceeded 685.5 μg/day were 7.3 times more likely (95% CI 5.1-10.3) to consume nitrates at the highest quartile of intake than participants with daily dietary folate intake < 319 μg/day.

### Nitrite intake

Compared to non-Hispanic White participants, non-Hispanic Black (OR 1.9; 95% CI 1.3-2.7), Hispanic (OR 6.2; 95% CI 4.3-9.0), and Asian/Pacific Islander participants (OR 10.3; 95% CI 5.4-19.6) were more likely to have an estimated nitrite consumption at the highest quartile of intake (Table 2). Participants with little or no education (0-8 years) were nearly 3 times more likely (95% CI 1.4-5.8) to consume dietary nitrites at the highest quartile of intake compared with participants with 16 or more years of education. Participants from Texas were more likely to have an estimated nitrite intake in the highest quartile compared with participants from other states. Participants whose fat intake exceeded 30% of their daily caloric intake were nearly 9 times (95% CI 6.9-11.0) more likely to consume more than 2.13 mg/day of dietary nitrite than those whose fat intake was less than 30% of their daily caloric intake. With adjustment of other maternal characteristics, participants who consumed more than 319 μg/day of dietary folate compared to participants with dietary folate consumption <319 μg/day were approximately twice as likely to have dietary nitrite consumption at the highest quartile of intake across the second through fourth quartiles of dietary folate intake.

### Total dietary nitrite intake

Compared with non-Hispanic White participants, non-Hispanic Black and Hispanic participants were approximately twice as likely to have total dietary nitrite consumption in the highest quartile of intake and Asian/Pacific Islander participants were approximately 9 times as likely (Table 3). Participants who completed 9-12 years of education were less likely to consume total dietary nitrites at the highest quartile of intake compared to those who completed 16 or more years. Also, Massachusetts and New Jersey participants were 1.7 times more likely to consume total dietary nitrites at the highest quartile of intake compared with those in Texas. In contrast, Utah participants were one-half (95% CI 0.29-0.90) as likely as Texas participants to have total dietary nitrite consumption at the highest quartile of intake.

An increase in dietary folate intake was significantly associated with an increase in total dietary nitrite intake. Participants who consumed more than 685.5 μg/day of dietary folate compared to participants with dietary folate consumption < 319 μg/day were 8.7 times (95% CI 6.0-12.6) more likely to also have total dietary nitrite consumption at the highest quartile of intake. Dietary fat intake greater than 30% of daily caloric intake was associated with greater than two-fold risk of total dietary nitrite intake at the highest quartile of intake (OR 2.5; 95% CI 2.0-3.0). Participants with a pre-pregnancy body mass index ≥ 30 were 1.8 times (95% CI 1.1-2.9) as likely as those of normal weight to have an estimated total nitrite exposure of more than 5.22 mg/day.

### Nitrosamine intake

Overall, non-Hispanic Black participants, Hispanic participants, and Asian/Pacific Islander participants were less likely to consume dietary nitrosamines in the highest quartile of intake than non-Hispanic White participants (Table 4). Compared to participants with 16 or more years of education, those with less education were less likely to consume nitrosamines at the highest quartile of intake. Compared with Texas participants, those from Iowa were 3.6 times (95% CI 2.4-5.5) more likely to have a nitrosamine intake in the highest quartile. In contrast, North Carolina participants were one-half as likely to be in the highest quartile of nitrosamine intake compared to those from Texas (OR 0.49; 95% CI 0.29-0.83). Participants whose daily dietary fat intake exceeded 30% of their daily caloric intake were 8.5 times (95% CI 6.9-10.4) more likely to have daily dietary nitrosamine intake of greater than 0.66 μg/day than participants with lower intake of dietary fat. Compared to dietary folate intake at the lowest quartile, increasing intake of dietary folate was associated with an intake of dietary nitrosamine at the highest quartile in the study population.

### Intake by food category

Table 5 displays the mean, median, and range of daily intake for nitrates, nitrites and nitrosamines for this study population. The values are presented as unadjusted values for comparison with other studies and as energy-adjusted values presented as per 1000 calories per day. Median unadjusted nitrate intake for non-Hispanic White participants was 37.02 mg/day compared with Asian/Pacific Islander, non-Hispanic Black, and Hispanic participants whose median nitrate intake was approximately 64.03 mg/day, 48.41 mg/day, and 45.29 mg/day respectively. Median consumption data for NBDPS control participants was calculated as 1.53 mg/day of dietary nitrite and 3.69 mg/day of total nitrite. Asian/Pacific Islander participants had the largest median intake of nitrates (64.03 mg/day) and total nitrites (5.07 mg/day). Hispanic participants had the highest unadjusted median intake of nitrites (2.04 mg/day). However, Asian/Pacific Islanders had the highest adjusted (for caloric intake) median intake of nitrites. Non-Hispanic White participants had the largest median nitrosamine intake (0.487 μg/day).

**Table 5:** Average daily intake dietary nitrates, nitrites and total nitrites by reported maternal race/ethnicity

	**Unadjusted**			**Adjusted**^**b**^		
		
	**Mean**^**a**^	**Median**	**Range**	**Mean**^**a**^	**Median**	**Range**
		
**Nitrate**	mg/day			mg/1000 calories/day		
White non-Hispanic	45.54 ± 34.72	37.02	3.01-638.44	31.97 ± 21.86	26.48	4.19-280.27
Black non-Hispanic	68.42 ± 65.62	48.41	5.39-692.23	42.09 ± 36.65	32.31	4.81-335.96
Hispanic	56.68 ± 43.23	45.29	2.51-382.06	29.23 ± 18.79	24.36	4.48-234.33
Asian/Pacific Islander	87.66 ± 79.36	64.03	8.03-556.12	54.61 ± 38.90	43.92	8.29-301.25
Other	60.42 ± 72.74	42.37	9.84-809.64	33.11 ± 22.08	27.83	7.37-175.05
All women	52.33 ± 45.60	40.84	2.51-809.64	33.21 ± 24.62	26.75	4.19-335.96
		
**Nitrite**	mg/day			mg/1000 calories/day		
White non-Hispanic	1.55 ± 0.85	1.40	0.12-26.51	1.08 ± 0.35	1.04	0.15-3.25
Black non-Hispanic	1.91 ± 1.27	1.64	0.20-13.53	1.13 ± 0.41	1.08	0.26-2.69
Hispanic	2.30 ± 1.38	2.04	0.19-19.78	1.18 ± 0.37	1.15	0.23-3.41
Asian/Pacific Islander	2.10 ± 1.51	1.67	0.10-12.23	1.28 ± 0.46	1.22	0.34-2.79
Other	1.94 ± 1.73	1.55	0.36-18.59	1.06 ± 0.35	1.05	0.26-2.10
All women	1.78 ± 1.14	1.53	0.10-26.51	1.11 ± 0.37	1.07	0.15-3.41
		
**Total nitrite**	mg/day			mg/1000 calories/day		
White non-Hispanic	3.83 ± 2.22	3.37	0.36-39.66	2.68 ± 1.22	2.45	0.36-15.65
Black non-Hispanic	5.34 ± 4.18	4.23	0.47-48.14	3.24 ± 2.03	2.76	0.56-18.58
Hispanic	5.13 ± 3.18	4.41	0.31-38.89	2.64 ± 1.09	2.49	0.45-13.08
Asian/Pacific Islander	6.48 ± 4.92	5.07	0.71-35.91	4.00 ± 2.12	3.45	0.82-17.24
Other	4.97 ± 5.11	3.84	1.12-59.07	2.71 ± 1.22	2.48	0.99-9.65
All women	4.41 ± 3.05	3.69	0.31-59.07	2.77 ± 1.37	2.51	0.35-18.58
		
**Nitrosamine**	μg/day			μg/1000 calories/day		
White non-Hispanic	0.548 ± .315	0.487	0.017-7.304	0.380 ± 0.157	0.361	0.014-1.945
Black non-Hispanic	0.534 ± .396	0.448	0.029-3.187	0.315+0.210	0.280	0.031-2.556
Hispanic	0.531 ± .348	0.463	0.050-3.878	0.278 ± 0.122	0.262	0.052-1.273
Asian/Pacific Islander	0.501 ± .386	0.413	0.009-2.852	0.305 ± 0.137	0.289	0.043-0.815
Other	0.507 ± .373	0.427	0.066-.3444	0.283 ± 0.117	0.267	0.044-0.738
All women	0.541 ± .337	0.472	0.009-7.304	0.344 ± 0.162	0.322	0.014-2.556

^a^Mean value ± the standard deviation

^b^Adjusted for daily caloric intake (values presented per 1000 calories consumed)

Intake of vegetable products for all participants accounted for approximately 61% of dietary nitrate intake, although intake of vegetables accounted for 74% of dietary nitrates among Asian/Pacific Islander participants (Additional File 2). The majority of dietary nitrite intake was attributed to meat (60.7%). Non-Hispanic Black participants received 67.9% of their daily dietary nitrite intake from this food group, which was more than non-Hispanic White participants, Hispanic participants, and Asian/Pacific Islander participants. The second largest food group contributing to dietary nitrite intake was grain products, contributing approximately 12% of dietary nitrite for all participants and more than 18% for Asian/Pacific Islander participants.

A main contributor to total dietary nitrite consumption was vegetables (40.5%) with meat contributing another 30.9%. Non-Hispanic White participants, non-Hispanic Black participants, Hispanic participants, and Asian/Pacific Islander participant's intake from vegetable products was 41.7%, 49.2%, 31.4% and 53.6% of their total dietary nitrite intake respectively. For meat and vegetable products combined, percentage of daily total nitrite intake was calculated as 74.1% for whites, 79.8% for blacks, 61.4% for Hispanics, and 76.3% for Asian/Pacific islanders.

Meat and dairy products contributed most of dietary nitrosamines for all participants (48.9% and 43.7% respectively). Approximately half of the dietary nitrosamine consumption for non-Hispanic White participants was attributed to dairy products (49.0%) compared to approximately 38% for Hispanic participants and Asian/Pacific Islander participants and 29.7% for non-Hispanic Black participants. In contrast, non-Hispanic Black participants (60.8%), Hispanic participants (54.5%), and Asian/Pacific Islander participants (57.1%) received more than half of their dietary nitrosamine intake from meat products compared to non-Hispanic White participants (44.2%). In this study population, alcohol was estimated to contribute less than 5% to daily nitrosamine intake.

### Most frequently consumed food items

We examined the average number of servings per day and the average daily intake of nitrates, nitrites, and nitrosamines by food item (Additional File 3). The top five foods most frequently consumed and the top five foods with the most substantial contributions to nitrate, nitrite, and nitrosamine intake varied by race/ethnicity. The five most frequently consumed food items are described as follows. Non-Hispanic white women most frequently consumed skim or lowfat milk, cereal, cheese, bread products, and orange juice. Non-Hispanic black women most frequently consumed cereal, bread products, whole milk, orange juice, and eggs. Hispanics reported tortillas, cereal, whole milk, orange juice, and fresh apples or pears as their most frequently ingested food. Asian/Pacific Islanders consumed rice or pasta, orange juice, cereal, skim milk, and fresh apples or pears most frequently.

The sources of nitrates, nitrites, and nitrosamines were explored by identifying the top five foods that contributed to the average daily intake by race/ethnicity. The foods that contribute the most to daily dietary nitrate, on average, for Non-Hispanic whites and blacks were spinach or collard greens, potatoes, broccoli, string beans, and orange juice. For Hispanics, the five foods contributing most to daily nitrate intake were spinach or collard greens, broccoli, potatoes, salsa, and orange juice. The five foods consumed contributing most to daily nitrate intake for Asian/Pacific Islanders were spinach or collard greens, broccoli, potatoes, cabbage (including cauliflower or Brussels sprouts), and rice or pasta.

The top five food items, starting with the largest, contributing to dietary nitrite intake for Non-Hispanic white women was beef (including pork, lamb or cabrito) as a main dish, beef (including pork, lamb, or cabrito) as a mixed dish, chicken or turkey, rice or pasta, and hot dogs. For non-Hispanic black women the top five contributors to dietary nitrite intake were beef (including pork, lamb or cabrito) as a main dish, chicken or turkey, beef (including pork, lamb or cabrito) a mixed dish, hot dogs, and bacon. Daily intake of dietary nitrites from beef (including pork, lamb or cabrito) as a main dish, refried beans, beef (including pork, lamb or cabrito) as a mixed dish, chicken or turkey, and rice or pasta were the top five contributors for Hispanics. The top five foods contributing to nitrite intake among Asian Pacific Islanders were beef (including pork, lamb or cabrito) as a main dish, rice or pasta, beef (including pork, lamb or cabrito) as a mixed dish, chicken or turkey, and fish.

The five foods consumed contributing most to daily dietary nitrosamine intake for non-Hispanic whites were cereal, skim or low fat milk, beef (including pork, lamb or cabrito,) as a main dish, beef (including pork, lamb or cabrito) as a mixed dish, and cheese. For non-Hispanic blacks, the top five foods were cereal, beef (including pork, lamb or cabrito) as a main dish, skim or low fat milk, bacon, and beef (including pork, lamb or cabrito) as a mixed dish. The top five foods contributing to daily nitrosamine intake for Hispanics were cereal, beef (including pork, lamb or cabrito) as main dish, skim or low fat milk, beef (including pork, lamb or cabrito) as a mixed dish, and whole milk. For Asian/Pacific Islanders, the top five foods with respect to daily nitrosamine intake were cereal, skim or low fat milk, beef (including pork, lamb or cabrito) as a main or mixed dish, and fish.

## Discussion

To our knowledge, this study is the first to describe maternal characteristics associated with estimated dietary intake of nitrates, nitrites, and nitrosamines for women of childbearing age. Factors associated with increased intake of these compounds are also related to risk of adverse pregnancy outcomes and other health conditions and, therefore, may be confounding factors in studies of the relation between these food contaminants and adverse health outcomes.

In this study, we found that estimated intake of nitrates, nitrites, and nitrosamines varied by several maternal characteristics. Reported maternal race and ethnicity, area of residence (state), and intake of folate were important predictors for consumption of these compounds. Minority women (non-Hispanic black, Hispanic, and Asian/Pacific islander) were more likely to consume greater amounts of nitrates, nitrites, and total nitrites compared with non-Hispanic white women. However, non-Hispanic white women consumed more dietary nitrosamines than women of other race/ethnic groups studied. Increased consumption of dietary nitrites and nitrosamines is generally considered unhealthy, and foods high in nitrite and nitrosamine content (processed meat, alcohol, dairy products) should be consumed in moderation. However, vegetables are the largest contributor to dietary nitrate and, in contrast to nitrites and nitrosamines, increased intake of vegetables is widely accepted as a healthy behavior associated with higher income, especially given the higher cost of fresh fruits and vegetables compared to less expensive processed foods. Increased consumption of vegetables at the highest quartile of intake would also increase folate consumption and other phyto-nutrients, reflecting a diet associated with the healthiest members of the population.

The median dietary nitrate and nitrite intake for women of child-bearing age included in this study was estimated at 40.84 mg/day and 1.53 mg/day respectively (energy-adjusted values, 26.75 and 1.07). Estimates of nitrate and nitrite intake from other populations have varied greatly, from 31 mg/day in Norway to 245 mg/day in Italy for nitrates and from 0.8 mg/day in the United States to 8.7 mg/day in Poland for nitrites [12,17,36]. Our estimates are within the range of published values of nitrate and nitrite intake for United States populations. Mensinga and colleagues reported the average total nitrate intake in the United States to be 40-100 mg/day [17]. White noted nitrate intake in the United States to be approximately 106 mg/day for nitrate and 4.1 mg/day for nitrite [37]. In a more recent and more closely related study, Brender and colleagues reported the median nitrate and nitrite intake for Mexican American women who resided in Texas counties along the Mexico border as 87 mg/day and 4.1 mg/day respectively [12]. In a study conducted in France by Menard and colleagues on nitrate and nitrite consumption from food and water, consumption data was collected from 1998-1999 for 1474 adults and 1018 children using a 7-day food frequency questionnaire [38]. Dietary nitrate intake was estimated at 1.5 mg/kg of body weight per day for adults and 1.9-2.0 mg/kg of body weight per day for children. Nitrite intake for adults was estimated at 0.02-0.04 mg/kg of body weight and 0.04-0.08 mg/kg of body weight for children. Based on the median pre-pregnancy body weight for control-women in the NBDPS (64 kg), average daily exposures from food and water using the French estimates would be approximately 96 mg for nitrate and 1.28-2.56 mg for nitrite. The median daily nitrate and nitrite intake solely from diet for our study population was estimated 40.48 mg and 1.53 mg respectively. Nitrosamine exposure from food and beverages was reported in 1981 by the National Academy of Sciences (NAS) at approximately 1.0 μg/day per capita [39]. Scanlan estimated nitrosamines at 0.1 μg/day because of the more recent efforts to prevent nitrosamine formation in foods and beverages [40]. Median dietary nitrosamine exposure for our study population was 0.472 μg/day (adjusted, 0.322 μg/day), larger than Scanlan's estimate yet smaller than the value reported by the NAS. It should be noted that while interesting to compare our results with those or other studies, the use of a food frequency questionnaire may not be appropriate for accurately quantifying intake without calibration, but can be used quite effectively to identify foods and consumption patterns that are likely to result in higher or lower exposure to dietary sources of nitrates, nitrites, and nitrosamines.

With adjustment for race/ethnicity, maternal age at conception and general or multi-vitamin use had little impact on estimates of consumption of these compounds and were excluded from all models. Although general or multivitamin supplementation may be used as a proxy for healthy behaviors in some instances, it may be more difficult to do so in a population of pregnant women. The source of vitamins is not documented and the potential differences in multivitamin use, especially those based on socioeconomic status, may be diminished for women who receive assistance from other sources such as Medicaid and the Women, Infants, and Children (WIC) program [41]. However, race/ethnicity, state or area of residence, and folate intake were important predictors for nitrate, nitrite, total nitrite, and nitrosamine intake in this study population. The prevalence of birth defects varies by race and ethnicity; likewise, the most common birth defect(s) experienced by a specific sub-population differs according to the racial/ethnic group considered [42].

Food choices and patterns of consumption also vary by race/ethnicity. Differences in food choices may yield differing exposures to dietary nitrosamines and their precursors. Intake of dietary folate equivalents, which account for differences in the absorption of naturally occurring food folate and the more bio-available synthetic folic acid, was an important predictor of intake in general, even after adjusting for caloric intake. Folate can be found naturally in a variety of foods such as lentils, meat and beans, fruits and vegetables; however, folic acid has also been added to enriched cereal grains and is now contained in hundreds of additional products [43,44]. The addition of folate to these products may account for the significant association with intake of these compounds; greater intake of food items in general may yield greater intake of dietary folate equivalents.

Based on our results, dietary nitrite intake for all races and ethnicities in this study population can be attributed largely to meat and bean products and grain products. Similar to nitrates, non-Hispanic whites in our study have lower intake of dietary nitrite and total nitrite, on average, than other race/ethnicities. Non-Hispanic black women had the highest average dietary nitrite and total nitrite from the meat category (1.3 and 1.6 mg/day). Asian/Pacific Islanders had the highest average intake of nitrite and total dietary nitrite from the vegetable (0.258 and 3.477 mg/day) and grain categories (0.394 and 0.540 mg/day). These findings seem to underscore the racial and ethnic differences in food choices.

In contrast to nitrate and nitrite consumption, Non-Hispanic white women consumed the most nitrosamines per day compared with participants of other race/ethnicities. Dairy products and meat and bean products contributed an estimated 93% of daily dietary nitrosamines, while alcohol accounted for only 2.4% of intake. Non-Hispanic white women consumed more nitrosamines per day from dairy products, on average, than did other women; whereas, non-Hispanic black women consumed more nitrosamines from meat products. However, the average daily amounts consumed from dairy products varied more than for meat products. Intake from dairy products ranged from 0.159-0.269 μg/day while average daily intake of nitrosamines from meat products ranged from 0.242-0.325 μg/day. When the two categories were combined, non-Hispanic white women consumed approximately 0.511 μg/day of dietary nitrosamines, more than non-Hispanic black women (0.484 μg/day), Hispanic women (0.496 μg/day), and Asian/Pacific Islanders (0.483 μg/day). The relatively low intake of nitrosamines among non-Hispanic black participants may be due to the lower contribution from dairy products. Lactose intolerance, perception that milk is for "children," having few role models who drink milk, and problems with transportation and storage have been documented barriers for milk consumption [45]. Milk and dairy products are the main sources of calcium in western countries [46]. A study conducted on data from the National Health and Nutrition Examination Survey from 1999-2002 found that 46.0% of non-Hispanic white men and women met the recommendation for adequate intake of calcium, compared to only 20.9% of non-Hispanic black and 33.4% of Mexican American participants [47]. The relatively high nitrosamine intake by race/ethnicity could be explained by the greater intake of nitrosamines from dairy products by non-Hispanic white women than women of other race/ethnicities in this population.

This study has several limitations. Estimated intake of nitrates, nitrites, and nitrosamines was restricted to dietary intake for each participant. For total nitrite (dietary nitrite + 5% dietary nitrate) it is important to note that vegetables are the main contributor for dietary nitrate, many of which are also rich in vitamin C--a vitamin known to inhibit the formation of nitrosamines under normal gastric conditions. Contributions of these compounds from drinking water, occupation, or environmental sources were not calculated, although estimates of nitrate intake from drinking water are currently being developed for NBDPS participants in Iowa and Texas. Average nitrate, nitrite, and nitrosamine content were assigned to items in the food frequency based on estimates of these compounds from published data that were mostly generated before 1990; therefore, estimates may not accurately represent more recent levels of these contaminants in foods [39]. The estimates are presented as two decimal places for nitrate, nitrites, and total nitrites and three decimal places for nitrosamines. The quartile breaks were established based on the actual distribution of participants and the number of decimal places reflects only the break between quartiles and should not be interpreted as an accurately measured value. The values are generated from estimates and, although useful for estimating consumption, may not be accurate representations of currently available food items.

Biases in recall and reporting may be an issue. Participants were asked to estimate their consumption of food items for the year before conception, and women might have selectively recalled food and alcohol intake based on race/ethnicity, income, body weight and other factors. However, this study examines only the information provided by NBDPS control women and any effect of recall bias should be minimal.

Estimates of nitrates, nitrites, and nitrosamines from food items were limited to the food items represented on the WFFQ. Broad designations of food groups such as "beef, pork, and lamb" and "fish" decreased accuracy of estimated nitrite and nitrosamine intake in two ways. First, estimates of nitrite and nitrosamine content in these food items were based on an average of these foods from data published in the literature including values for fresh, smoked, and pickled items. Second, the intake of fresh or cured items could not be distinguished among participants. These limitations most likely led to overestimation of nitrite and nitrosamine intake among women who reported consumption of these food items.

This questionnaire also may not capture all potential sources of nitrate, nitrite, and nitrosamine exposures nor accurately reflect culturally appropriate foods. Our study had relatively few Native Americans (n = 27) and Asian/Pacific Islander participants (n = 176) overall, and their distribution differed greatly by area of residence. Another potential limitation is that we did not adjust for multiple comparisons in our observational study. Adjusting for multiple comparisons has been heavily debated in the literature. Rothman posits it might be preferable to not adjust for multiple comparisons "because it will lead to fewer errors of interpretation when the data under evaluation are not random numbers but actual observations in nature" [48].

This study has several strengths as well. Estimates of nitrate, nitrite, and nitrosamine intake were based on the 5,958 controls participating in the National Birth Defects Prevention Study, a population-based, case-control study that involves 10 states. Characteristics of women of child-bearing age that are associated with intake of nitrates, nitrites, total nitrites, and nitrosamines have not been well-described in the literature.

## Conclusion

Results of this study indicate that intake of nitrates, nitrites, and nitrosamines vary considerably by race/ethnicity and other characteristics. Study findings may be used to generate hypotheses for further research on the consumption of nitrosamines and *N*-nitroso precursors in populations and relation of this dietary consumption to risk for adverse reproductive outcomes, cancer, and cardiovascular disease. Future studies should focus on identifying the dietary contribution of nitrates, nitrites, and nitrosamines from specific food items commonly consumed in various populations. Estimates of exposure and dietary patterns of intake of nitrates, nitrites, and nitrosamines by race and ethnicity and other important factors including culturally relevant food items should be considered as future areas of research. Differences in dietary intake of these compounds may have a role in causal pathways for adverse reproductive outcomes and chronic diseases such as cancer in women.

## Abbreviations

In this paper, the following abbreviations were used: (BIC): Bayesian Information Criteria; (CDC): Centers for Disease Control and Prevention; (CI): confidence interval; (DFE): dietary folate equivalents; (EDD): estimated date of delivery; (μg): micrograms; (mg): milligrams; (NBDPS): National Birth Defects Prevention Study; (OR): odds ratio; (USDA): United States Department of Agriculture; (WFFQ): Willet Food Frequency Questionnaire.

## Competing interests

The authors declare that they have no competing interests.

## Authors' contributions

JG carried out the literature review; assigned the daily nitrate, nitrite, and nitrosamine intake of the participants; conducted the data analysis; and prepared the manuscript. JB conceived of the study; developed the methods for dietary nitrate, nitrite, and nitrosamine assignment; reviewed the data analyses; and contributed to the abstract, results and discussion. JS contributed to the nutritional aspects and discussion section of the manuscript. MS assisted with assignment of daily nitrate, nitrite, and nitrosamine intake of the participants and reviewed the data analyses. JH assisted with the statistical analyses and interpretation of the data. AR and TM provided input regarding the methods and contributed to the discussion. PR helped develop methods for dietary nitrate, nitrite, and nitrosamine assignment and provided input into maternal factors to be considered in analyses. MC, PL, and LS helped develop the methods for dietary nitrate, nitrite, and nitrosamine assignment and contributed to the discussion. All authors read and approved the final manuscript.

## Supplementary Material

Additional file 1 **Odds ratios and 95% confidence intervals for all quartiles of nitrate, nitrites, total nitrites, and nitrosamines**. These tables show maternal characteristics associated with intake of nitrates, nitrites, total nitrites, and nitrosamines by quartile of intake. Crude and adjusted odds ratios are presented. Click here for file

Additional file 2 **Contributions of food groups to nitrate, nitrite, total nitrite, and nitrosamine intake**. These tables show the estimated contribution and percent contribution of food groups to nitrate, nitrite, total nitrite, and nitrosamine intake by race/ethnicity. Click here for file

Additional file 3 **Contributions of individual food items to average daily nitrite, nitrite and nitrosamine intake by race/ethnicity**. This table shows the average nitrate, nitrite, and nitrosamine contribution per day from each food item on the National Birth Defects Prevention Study food frequency questionnaire by race/ethnicity. Click here for file
