# Supplementary material for: Maternal characteristics associated with the dietary intake of nitrates, nitrites, and nitrosamines in women of child-bearing age: a cross-sectional study
Source: Environ Health. 2010 Feb 19;9:10. doi: 10.1186/1476-069X-9-10 (PMC2848640; doi:10.1186/1476-069X-9-10)
Supplement: Additional file 1 — Odds ratios and 95% confidence intervals for all quartiles of nitrate, nitrites, total nitrites, and nitrosamines. These tables show maternal characteristics associated with intake of nitrates, nitrites, total nitrites, and nitrosamines by quartile of intake. Crude and adjusted odds ratios are presented. [file 1476-069X-9-10-S1.DOC]

Additional File 1

Odds Ratios and 95% Confidence Intervals for all Quartiles of Intake of Nitrates, Nitrites, Total Nitrites, and Nitrosamines

| **Table F1A: Odds ratios and 95% confidence intervals for dietary nitrate intake (mg/day) by maternal characteristics** | | | | | | | | | | | | | | | | | |
| --- | --- | --- | --- | --- | --- | --- | --- | --- | --- | --- | --- | --- | --- | --- | --- | --- | --- |
|  | **Quartile 1^a^ 2.509-**  **27.059** | | | | | **Quartile 2 27.060-40.485** | | **Odds Ratios and 95% confidence intervals** | | **Quartile 3 40.486-60.602** | | **Odds Ratios and 95% confidence intervals** | | **Quartile 4 60.603-**  **809.64** | | **Odds Ratios and 95% confidence intervals** | |
|  | n | | | | % | **n** | % | **Crude** | **Adjusted^b^** | **n** | **%** | **Crude** | **Adjusted^b^** | **n** | **%** | **Crude** | **Adjusted^b^** |
| **Race/Ethnicity** |  | | | |  |  |  |  |  |  |  |  |  |  |  |  |  |
| White non-Hispanic | 991 | | | | 28.4 | 964 | 27.6 | 1.0 | 1.0 | 890 | 25.5 | 1.0 | 1.0 | 647 | 18.5 | 1.0 | 1.0 |
| Black non-Hispanic | 130 | | | | 19.7 | 119 | 18.1 | 0.94 (0.72,1.2) | 0.98 (0.71,1.3) | 167 | 25.3 | **1.4 (1.1,1.8)** | **1.5 (1.1,2.0)** | 243 | 36.9 | **2.9 (2.3,3.6)** | **2.4 (1.7,3.3)** |
| Hispanic | 267 | | | | 20.6 | 292 | 22.6 | 1.1 (0.93,1.4) | 1.0 (0.78,1.4) | 318 | 24.6 | **1.3 (1.1,1.6)** | 1.0 (0.77,1.4) | 417 | 32.2 | **2.4 (2.0,2.9)** | 1.4 (1.0,1.9) |
| Asian/Pacific Islander | 25 | | | | 14.9 | 19 | 11.3 | 0.78 (0.43,1.4) | 0.85 (0.44,1.6) | 36 | 21.4 | 1.6 (0.95,2.7) | 1.5 (0.83,2.8) | 88 | 52.4 | **5.4 (3.4,8.5)** | **4.8 (2.7,8.5)** |
| Other | 34 | | | | 19.4 | 51 | 29.1 | 1.5 (0.99,2.4) | 1.5 (0.89,2.6) | 39 | 22.3 | 1.3 (0.80,2.0) | 1.1 (0.64,2.1) | 51 | 29.1 | **2.3 (1..5,3.6)** | 1.6 (0.89,2.9) |
| **State/Area of residence** |  | | | |  |  |  |  |  |  |  |  |  |  |  |  |  |
| Texas | 168 | | | | 24.5 | 192 | 28.0 | 1.0 | 1.0 | 165 | 24.0 | 1.0 | 1.0 | 162 | 23.6 | 1.0 | 1.0 |
| Arkansas | 194 | | | | 27.5 | 182 | 25.8 | 0.82 (0.61,1.1) | 0.72 (0.51,1.0) | 168 | 23.8 | 0.88 (0.65,1.2) | 0.74 (0.51,1.1) | 161 | 22.8 | 0.86 (0.64,1.2) | 0.79 (0.52,1.2) |
| California | 191 | | | | 24.5 | 165 | 21.2 | 0.76 (0.56,1.0) | **0.71 (0.51,0.99)** | 197 | 25.3 | 1.1 (0.78,1.4) | 0.94 (0.66,1.3) | 226 | 29.0 | 1.2 (0.92,1.6) | 0.98 (0.68,1.4) |
| Iowa | 245 | | | | 37.9 | 187 | 29.0 | **0.67 (0.50,0.88)** | **0.64 (0.45,0.90)** | 147 | 22.8 | **0.61 (0.45,0.82)** | **0.58 (0.39,0.85)** | 67 | 10.4 | **0.30 (0.20,.0.40)** | 0.37 (0.23,1.4) |
| Massachusetts | 150 | | | | 20.2 | 192 | 25.9 | 1.1 (0.83,1.5) | 1.4 (0.94,1.9) | 216 | 29.1 | **1.5 (1.1,2.0)** | 2.0 (1.4,2.9) | 184 | 24.8 | 1.3 (0.94,1.7) | **2.9 (1.9,4.5)** |
| New Jersey | 103 | | | | 18.2 | 122 | 21.6 | 1.0 (0.74,1.5) | 1.3 (0.84,1.9) | 152 | 26.9 | 1.5 (1.0,2.1) | 1.8 (1.2,2.7) | 189 | 33.4 | 1.9 (1.4,2.6) | **2.7 (1.7,4.1)** |
| New York | 127 | | | | 24.2 | 126 | 24.1 | 0.87 (0.63,1.2) | 1.0 (0.69,1.5) | 135 | 25.8 | 1.1 (0.78,1.5) | 1.4 (0.91,2.1) | 136 | 26.0 | 1.1 (0.80,1.5) | **1.8 (1.1,2.8)** |
| Georgia | 129 | | | | 21.2 | 127 | 20.8 | 0.86 (0.63,1.2) | 1.1 (0.74,1.6) | 165 | 27.1 | 1.3 (0.95,1.8) | 1.6 (1.1,2.5) | 189 | 31.0 | 1.5 (1.1,2.1) | **2.2 (1.4,3.4)** |
| North Carolina | 72 | | | | 24.7 | 81 | 27.7 | 0.98 (0.67,1.4) | 1.1 (0.72,1.7) | 54 | 18.5 | 0.76 (0.51,1.2) | 0.84 (0.51,1.4) | 85 | 29.1 | 1.2 (0.84,1.8) | **1.7 (1.1,2.9)** |
| Utah | 74 | | | | 28.7 | 78 | 30.2 | 0.92 (0.63,1.4) | 0.80 (0.52,1.2) | 53 | 20.5 | 0.73 (0.48,1.1) | 0.69 (0.42,1.1) | 53 | 20.5 | 0.74 (0.49,1.1) | 0.99 (0.59,1.7) |
| **Maternal household income (dollars annually)** | | | | | | |  |  |  |  |  |  |  |  |  |  |  |
| >50,000 | | 442 | | 24.1 | | 469 | 25.6 | 1.0 | 1.0 | 496 | 27 | 1.0 | 1.0 | 428 | 23.3 | 1.0 | 1.0 |
| 40,000-49,999 | | 120 | | 28.4 | | 114 | 27.0 | 0.90 (0.67,1.2) | 0.89 (0.66,1.2) | 100 | 23.6 | 0.74 (0.55,1.0) | **0.69 (0.50,0.96)** | 89 | 21.0 | 0.77 (0.56,1.0) | **0.67 (0.47,0.97)** |
| 30,000-39,999 | | 138 | | 25.5 | | 153 | 28.3 | 1.0 (0.80,1.4) | 1.0 (0.75,1.3) | 134 | 24.8 | 0.87 (0.66,1.1) | 0.77 (0.57,1.1) | 116 | 21.4 | 0.87 (0.66,1.1) | 0.71 (0.50,1.0) |
| 20,000-29,999 | | 180 | | 24.2 | | 203 | 27.3 | 1.1 (0.84,1.3) | 0.94 (0.72,1.2) | 182 | 24.4 | 0.90 (0.71,1.1) | **0.72 (0.55,0.96)** | 180 | 24.2 | 1.0 (0.81,1.3) | **0.71 (0.52,0.97)** |
| 10,000-10,999 | | 165 | | 22.9 | | 167 | 23.1 | 0.95 (0.74,1.2) | 0.73 (0.55,0.97) | 189 | 26.2 | 1.0 (0.80,1.3) | **0.59 (0.43,0.79)** | 201 | 27.8 | 1.3 (0.98,1.6) | **0.49 (0.36,0.68)** |
| <10,000 | | 233 | | 25.2 | | 205 | 22.1 | 0.83 (0.66,1.0) | 0.62 (0.47,0.81) | 198 | 21.4 | 0.76 (0.60,0.95) | **0.39 (0.29,0.53)** | 290 | 31.3 | 1.3 (1.0,1.6) | **0.43 (0.32,0.59)** |
| **Dietary folate equivalent (µg/day)** | | |  | | |  |  |  |  |  |  |  |  |  |  |  |  |
| <=318.91 | | 725 | | 50.4 | | 422 | 29.4 | 1.0 | 1.0 | 202 | 14.1 | 1.0 | 1.0 | 89 | 6.2 | 1.0 | 1.0 |
| >318.91<=464.92 | | 351 | | 24.0 | | 463 | 31.7 | **2.3 (1.9,2.7)** | **1.7 (1.3,2.0)** | 416 | 28.4 | **4.3 (3.4,5.3)** | **2.9 (2.3,3.6)** | 233 | 15.9 | **5.4 (4.1,7.1)** | **3.4 (2.5,4.7)** |
| >464.92<=685.31 | | 232 | | 15.9 | | 350 | 23.9 | **2.6 (2.1,3.2)** | **1.4 (1.1,1.8)** | 451 | 30.9 | **7.0 (5.6,8.7)** | **2.9 (2.2,3.8)** | 429 | 29.3 | **15.1 (11.5,19.8)** | **5.5 (4.0,7.6)** |
| >685.31 | | 145 | | 10.0 | | 217 | 15.0 | **2.6 (2.0,3.3)** | **1.0 (0.77,1.4)** | 383 | 26.5 | **9.5 (7.4,12.1)** | **2.5 (1.8,3.4)** | 701 | 48.5 | **39.4 (29.7,52.3)** | **7.3 (5.1,10.3)** |
| ^a^Referent category is nitrate intake at the lowest quartile of intake (<27.06 mg/day) | | | | | | | | | |  |  |  |  |  |  |  |  |
| ^b^Adjusted for tertiles of daily caloric intake, race/ethnicity, state of residence, maternal household income, and dietary folate as dietary folate equivalents. | | | | | | | | | | | | | | | | | |

| **Table F1B: Odds ratios and 95% confidence intervals for dietary nitrite intake (mg/day) by maternal characteristics** | | | | | | | | | | | | | | |
| --- | --- | --- | --- | --- | --- | --- | --- | --- | --- | --- | --- | --- | --- | --- |
|  | **Quartile 1^a^  0.10123-**  **1.12224** | | **Quartile 2 1.12225- 1.53386** | | **Odds Ratios and 95% confidence intervals** | | **Quartile 3 1.53387-2.13050** | | **Odds Ratios and 95% confidence intervals** | | **Quartile 4 2.13051-26.51469** | | **Odds Ratios and 95% confidence intervals** | |
|  | **n** | **%** | **n** | **%** | **Crude** | **Adjusted^b^** | **n** | **%** | **Crude** | **Adjusted^b^** | **n** | **%** | **Crude** | **Adjusted^b^** |
| **Race/Ethnicity** |  |  |  |  |  |  |  |  |  |  |  |  |  |  |
| White non-Hispanic | 1044 | 29.9 | 1016 | 29.1 | 1.0 | 1.0 | 889 | 25.4 | 1.0 | 1.0 | 548 | 15.7 | 1.0 | 1.0 |
| Black non-Hispanic | 155 | 23.5 | 138 | 20.9 | 0.91 (0.72,1.2) | 1.0 (0.77,1.4) | 182 | 27.6 | **1.4 (1.1,1.7)** | **1.5 (1.1, 2.1)** | 185 | 28 | **2.3 (1.8,2.9)** | **1.9 (1.3,2.7)** |
| Hispanic | 166 | 12.8 | 214 | 16.5 | **1.3 (1.1,1.7)** | **1.6 (1.2,2.2)** | 305 | 23.6 | **2.2 (1.8,2.7)** | **2.3 (1.6,3.2)** | 610 | 47.1 | **7.0 (5.7,8.6)** | **6.2 (4.3,9.0)** |
| Asian/Pacific Islander | 33 | 19.5 | 42 | 24.9 | 1.3 (0.82,2.1) | **1.9 (1.2,3.3)** | 36 | 21.3 | 1.3 (0.80,2.1) | **2.6 (1.5,4.7)** | 58 | 34.3 | **3.3 (2.2,5.2)** | **10.3 (5.4,19.6)** |
| Other | 45 | 25.7 | 40 | 22.9 | 0.91 (0.59,1.4) | 1.1 (0.65,1.9) | 40 | 22.9 | 1.0 (0.68,1.7) | 1.2 (0.69,2.2) | 50 | 28.6 | **2.1 (1.4,3.2)** | **2.2 (1.2,4.2)** |
| **Maternal education (years completed)** | | |  |  |  |  |  |  |  |  |  |  |  |  |
| 16+ | 538 | 29.4 | 585 | 31.9 | 1.0 | 1.0 | 456 | 24.9 | 1.0 | 1.0 | 251 | 13.7 | 1.0 | 1.0 |
| 13-15 | 374 | 24.3 | 388 | 25.2 | **0.95 (0.80,1.1)** | 0.95 (0.76,1.2) | 428 | 27.8 | **1.4 (1.1,1.6)** | 1.2 (0.92,1.5) | 352 | 22.8 | **2.0 (1.6,2.5)** | 1.3 (0.98,1.8) |
| 12 | 356 | 24.7 | 316 | 21.9 | **0.82 (0.67,0.99)** | 0.82 (0.63,1.1) | 332 | 23 | **1.1 (0.91,1.3)** | 0.82 (0.62,1.1) | 438 | 30.4 | **2.6 (2.1,3.2)** | 1.2 (0.88,1.7) |
| 9-11 | 145 | 20.8 | 112 | 16.1 | **0.71 (0.54,0.93)** | 0.59 (0.41,0.86) | 171 | 24.5 | **1.4 (1.1,1.8)** | 0.89 (0.60,1.3) | 269 | 38.6 | **4.0 (3.1,5.1)** | 1.2 (0.79,1.9) |
| 0-8 | 31 | 11.2 | 47 | 16.9 | 1.4 (0.87,2.3) | 1.4 (0.75,2.7) | 63 | 22.7 | **2.4 (1.5,3.8)** | **2.0 (1.0,3.8)** | 136 | 49.1 | **9.4 (6.2,14.3)** | **2.9 (1.4,5.8)** |
| **State/Area of residence** |  |  |  |  |  |  |  |  |  |  |  |  |  |  |
| Texas | 106 | 18.6 | 128 | 18.6 | 1.0 | 1.0 | 171 | 24.9 | 1.0 | 1.0 | 282 | 41.1 | 1.0 | 1.0 |
| Arkansas | 145 | 21.5 | 152 | 21.5 | 0.87 (0.62,1.2) | 0.83 (0.55,1.2) | 196 | 27.7 | 0.84 (0.61,1.2) | 0.82 (0.54,1.3) | 215 | 30.4 | **0.56 (0.41,0.76)** | 0.88 (0.55,1.4) |
| California | 149 | 18.5 | 145 | 18.5 | 0.81 (0.57,1.1) | 0.68 (0.46,1.0) | 199 | 25.5 | **0.83 (0.60,1.1)** | **0.65 (0.43,0.97)** | 289 | 37.0 | **0.73 (0.54,0.98)** | **0.49 (0.32,0.76)** |
| Iowa | 184 | 24.0 | 155 | 24.0 | **0.70 (0.50,0.97)** | **0.67 (0.45,0.99)** | 171 | 26.5 | **0.58 (0.42,0.79)** | 0.67 (0.44,1.0) | 136 | 21.1 | **0.28 (0.20,0.38)** | 0.67 (0.42,1.1) |
| Massachusetts | 234 | 35.6 | 264 | 35.6 | 0.93 (0.68,1.3) | 0.86 (0.59,1.3) | 175 | 23.6 | **0.46 (0.34,0.63)** | **0.55 (0.36,0.83)** | 69 | 9.3 | **0.11 (0.08,0.16)** | **0.30 (0.18,0.50)** |
| New Jersey | 156 | 25.6 | 145 | 25.6 | 0.77 (0.55,1.1) | 0.73 (0.49,1.1) | 135 | 23.9 | **0.54 (0.38,0.75)** | **0.61 (0.39,0.94)** | 130 | 23.0 | **0.31 (0.23,0.43)** | **0.50 (0.30,0.82)** |
| New York | 161 | 27.2 | 143 | 27.2 | 0.74 (0.52,1.0) | 0.77 (0.51,1.2) | 125 | 23.8 | **0.48 (0.34,0.67)** | **0.56 (0.36,0.88)** | 96 | 18.3 | **0.22 (0.16,0.3)** | **0.51 (0.31,0.86)** |
| Georgia | 165 | 25.3 | 155 | 25.3 | 0.78 (0.55,1.1) | 0.83 (0.55,1.3) | 153 | 25.0 | **0.57 (0.41,0.80)** | **0.61 (0.39,0.95)** | 139 | 22.7 | **0.32 (0.23,0.43)** | **0.54 (0.33,0.89)** |
| North Carolina | 74 | 25.3 | 87 | 29.8 | 0.97 (0.65,1.5) | 1.0 (0.65,1.7) | 73 | 25.0 | **0.61 (0.41,0.92)** | 0.71 (0.42,1.2) | 58 | 19.9 | **0.29 (0.20,0.44)** | **0.45 (0.24,0.82)** |
| Utah | 81 | 31.4 | 80 | 31.0 | 0.82 (0.55,1.2) | 0.64 (0.41,1.0) | 57 | 22.1 | **0.44 (0.29,0.66)** | **0.33 (0.20,0.55)** | 40 | 15.5 | **0.19 (0.12,0.29)** | **0.22 (0.12,0.41)** |
| **Dietary fat intake (% of daily calories)** | | |  |  |  |  |  |  |  |  |  |  |  |  |
| < 30% | 950 | 29.4 | 827 | 25.6 | 1.00 | 1.0 | 756 | 23.4 | 1.0 | 1.0 | 702 | 21.7 | 1.0 | 1.0 |
| >30% | 505 | 19.5 | 627 | 24.3 | **1.4 (1.2,1.7)** | **2.1 (1.8,2.6)** | 699 | 27.1 | **1.7 (1.5,2.0)** | **3.7 (3.1,4.5)** | 752 | 29.1 | **2.0 (1.7,2.3)** | **8.7 (6.9,11.0)** |
| **Maternal household income (dollars annually)** | | | |  |  |  |  |  |  |  |  |  |  |  |
| >50,000 | 559 | 30.5 | 582 | 31.2 | 1.0 | 1.0 | 452 | 24.6 | 1.0 | 1.0 | 243 | 13.2 | 1.0 | 1.0 |
| 40,000-49,999 | 117 | 27.7 | 119 | 28.1 | 0.98 (0.74,1.3) | 1.1 (0.79,1.5) | 106 | 25.1 | 1.1 (0.84,1.5) | 1.0 (0.73,1.5) | 81 | 19.2 | **1.6 (1.2,2.2)** | 1.0 (0.66,1.6) |
| 30,000-39,999 | 138 | 25.5 | 130 | 24.0 | 0.90 (0.69,1.2) | 0.97 (0.72,1.3) | 143 | 26.4 | 1.3 (0.98,1.7) | 1.1 (0.83,1.6) | 131 | 24.2 | **2.2 (1.6,2.9)** | 1.4 (0.92,2.0) |
| 20,000-29,999 | 168 | 22.6 | 178 | 23.9 | 1.0 (0.80,1.3) | 1.0 (0.77,1.4) | 188 | 25.2 | **1.4 (1.1,1.8)** | 1.0 (0.7,1.4) | 211 | 28.3 | **2.9 (2.2,3.7)** | 1.2 (0.81,1.7) |
| 10,000-10,999 | 132 | 18.3 | 133 | 18.4 | 0.97 (0.74,1.3) | 0.91 (0.66,1.3) | 192 | 26.6 | **1.8 (1.4,2.3)** | 1.0 (0.73,1.5) | 266 | 36.8 | **4.6 (3.6,6.0)** | 1.0 (0.68,1.5) |
| <10,000 | 196 | 21.1 | 166 | 17.8 | 0.81 (0.64,1.0) | 0.76 (0.55,1.0) | 201 | 21.6 | 1.3 (1.0,1.6) | **0.63 (0.44,0.90)** | 368 | 39.5 | **4.3 (3.4,5.4)** | 0.71 (0.48,1.1) |
| **Dietary folate equivalent (µg/day)** | | |  |  |  |  |  |  |  |  |  |  |  |  |
| <319 | 670 | 46.5 | 398 | 27.6 | 1.0 | 1.0 | 267 | 18.5 | 1.0 | 1.0 | 105 | 7.3 | 1.0 | 1.0 |
| 319-464.9 | 369 | 25.2 | 440 | 30.0 | **2.0 (1.7,2.4)** | **1.7 (1.4,2.1)** | 410 | 28.0 | **2.8 (2.3,3.4)** | **1.9 (1.5,2.4)** | 246 | 16.8 | **4.3 (3.3,5.5)** | **1.9 (1.3,2.6)** |
| 465-685.5 | 267 | 18.2 | 374 | 25.5 | **2.4 (1.9,2.9)** | **1.6 (1.3,2.1)** | 430 | 29.4 | **4.0 (3.3,5.0)** | **1.8 (1.4,2.4)** | 394 | 26.9 | **9.4 (7.3,12.2)** | **1.8 (1.3,2.6)** |
| >685.5 | 149 | 10.3 | 242 | 16.7 | **2.7 (2.2,3.5)** | **1.5 (1.1,2.0)** | 348 | 24.0 | **5.9 (4.6,7.4)** | **1.5 (1.1,2.2)** | 709 | 49.0 | **30.4 (23.2,39.8)** | **2.3 (1.5,3.3)** |
| ^a^Referent category is nitrite intake at the lowest quartile of intake (<1.12 mg/day) | | | | | | |  |  |  |  |  |  |  |  |
| ^b^Adjusted for tertiles of daily caloric intake, race/ethnicity, maternal education, state of residence, dietary fat intake, maternal household income, and dietary folate as dietary folate equivalents. | | | | | | | | | | | | | | |

| **Table F1C: Odds ratios and 95% confidence intervals for total dietary nitrite intake (mg/day) by maternal characteristics** | | | | | | | | | | | | | | |
| --- | --- | --- | --- | --- | --- | --- | --- | --- | --- | --- | --- | --- | --- | --- |
|  | **Quartile 1^a^ 0.31362-2.63578** | | **Quartile 2 2.63579-**  **3.69108** | | **Odds Ratios and 95% confidence intervals** | | **Quartile 3 3.69109-**  **5.21853** | | **Odds Ratios and 95% confidence intervals** | | **Quartile 4 5.21854-**  **59.07172** | | **Odds Ratios and 95% confidence intervals** | |
|  | **n** | **%** | **n** | **%** | **Crude** | **Adjusted^b^** | **n** | **%** | **Crude** | **Adjusted^b^** | **n** | **%** | **Crude** | **Adjusted^b^** |
| **Race/Ethnicity** |  |  |  |  |  |  |  |  |  |  |  |  |  |  |
| White non-Hispanic | 1024 | 29.3 | 988 | 28.3 | 1.0 | 1.0 | 903 | 25.9 | 1.0 | 1.0 | 577 | 16.5 | 1.0 | 1.0 |
| Black non-Hispanic | 132 | 20.0 | 134 | 20.3 | 1.1 (0.81,1.4) | 1.1 (0.82,1.5) | 151 | 22.9 | 1.3 (1.0,1.7) | 1.2 (0.90,1.7) | 242 | 36.7 | **3.3 (2.6,4.1)** | **2.6 (1.8,3.6)** |
| Hispanic | 222 | 17.2 | 263 | 20.3 | **1.2 (1.0,1.5)** | 1.1 (0.83,1.5) | 315 | 24.3 | **1.6 (1.3,2.0)** | 1.1 (0.83,1.5) | 494 | 38.2 | **3.9 (3.3,4.8)** | **2.0 (1.5,2.8)** |
| Asian/Pacific Islander | 28 | 16.7 | 19 | 11.3 | 0.70 (0.39,1.3) | 1.0 (0.55,1.9) | 39 | 23.2 | 1.6 (0.96,2.6) | **2.8 (1.5,4.9)** | 82 | 48.8 | **5.2 (3.3, 8.1)** | **9.2 (5.1,16.7)** |
| Other | 39 | 22.3 | 42 | 24.0 | 1.1 (0.72,1.7) | 1.3 (0.76,2.1) | 43 | 24.6 | 1.3 (0.80,1.9) | 1.2 (0.67,2.1) | 51 | 29.1 | **2.3 (1.5,3.6)** | **1.9 (1.1,3.5)** |
| **Maternal education (years completed)** | | |  |  |  |  |  |  |  |  |  |  |  |  |
| 16+ | 473 | 25.9 | 517 | 28.3 | 1.0 | 1.0 | 458 | 25 | 1.0 | 1.0 | 381 | 20.8 | 1.0 | 1.0 |
| 13-15 | 390 | 25.3 | 393 | 25.5 | 0.92 (0.76,1.1) | 0.88 (0.72,1.1) | 400 | 26 | 1.1 (0.88,1.3) | 1.0 (0.79,1.3) | 357 | 23.2 | 1.1 (093,1.4) | 0.80 (0.61,1.0) |
| 12 | 377 | 26.2 | 346 | 24.1 | 0.84 (0.69,1.0) | **0.71 (0.57,0.88)** | 349 | 24.3 | 0.96 (0.79,1.2) | **0.73 (0.57,0.92)** | 366 | 25.5 | 1.2 (0.99,1.5) | **0.55 (0.42,0.73)** |
| 9-11 | 164 | 23.5 | 142 | 20.4 | 0.79 (0.61,1.0) | **0.61 (0.45,0.83)** | 163 | 23.4 | 1.0 (0.79,1.3) | **0.61 (0.44,0.86)** | 228 | 32.7 | **1.7 (1.4,2.2)** | **0.49 (0.34,0.70)** |
| 0-8 | 42 | 15.2 | 46 | 16.7 | 1.0 (0.65,1.6) | 0.82 (0.45,1.5) | 74 | 26.8 | **1.8 (1.2,2.7)** | 0.97 (0.51,1.8) | 114 | 41.3 | **3.4 (2.3,4.9)** | 0.87 (0.46,1.7) |
| **Dietary folate equivalent (µg/day)** | |  |  |  |  |  |  |  |  |  |  |  |  |  |
| <319 | 739 | 51.4 | 427 | 29.7 | 1.0 | 1.0 | 208 | 14.5 | 1.0 | 1.0 | 64 | 4.5 | 1.0 | 1.0 |
| 319-464.9 | 349 | 23.9 | 463 | 31.7 | **2.3 (1.9,2.8)** | **1.8 (1.5,2.2)** | 441 | 30.1 | **4.5 (3.6,5.2)** | **2.8 (2.3,3.6)** | 210 | 13.4 | **6.9 (5.1,9.4)** | **4.0 (2.9,5.7)** |
| 465-685.5 | 232 | 15.9 | 352 | 24.1 | **2.6 (2.1,3.2)** | **1.7 (1.3,2.1)** | 452 | 30.9 | **6.9 (5.6,8.6)** | **2.8 (2.2,3.6)** | 426 | 29.1 | **21.2 (15.7,28.7)** | **6.4 (4.5,9.1)** |
| >685.5 | 133 | 9.2 | 210 | 14.5 | **2.7 (2.1,3.5)** | 1.3 (0.98,1.8) | 351 | 24.3 | **9.4 (7.3,12.1)** | **2.3 (1.7,3.1)** | 752 | 52 | **65.3 (47.6,89.5)** | **8.7 (6.0,12.6)** |
| **State/Area of residence** |  |  |  |  |  |  |  |  |  |  |  |  |  |  |
| Texas | 140 | 20.4 | 171 | 24.9 | 1.0 | 1.0 | 166 | 24.2 | 1.0 | 1.0 | 210 | 30.6 | 1.0 | 1.0 |
| Arkansas | 178 | 25.3 | 181 | 25.7 | 0.83 (0.61,1.1) | 0.76 (0.53,1.1) | 163 | 23.1 | 0.77 (0.57,1.1) | 0.73 (0.49,1.1) | 183 | 26.0 | **0.69 (0.51,0.93)** | 0.76 (0.50,1.2) |
| California | 176 | 22.6 | 162 | 20.8 | 0.75 (0.55,1.0) | 0.71 (0.50,1.0) | 188 | 24.1 | 0.90 (0.66,1.2) | 0.81 (0.56,1.2) | 253 | 32.5 | 0.96 (0.72,1.3) | 0.84 (0.57,1.2) |
| Iowa | 229 | 35.5 | 178 | 27.6 | **0.64 (0.47,0.86)** | **0.60 (0.42,0.85)** | 155 | 24.0 | **0.57 (0.42,0.77)** | **0.59 (0.40,0.87)** | 84 | 13.0 | **0.24 (0.18,0.34)** | 0.40 (0.25,0.63) |
| Massachusetts | 173 | 23.3 | 213 | 28.7 | 1.0 (0.75,1.4) | 1.3 (0.87,1.8) | 215 | 28.9 | 1.0 (0.78,1.4) | 1.8 (1.2,2.7) | 141 | 19.0 | **0.54 (0.40,0.74)** | **1.7 (1.1,2.7)** |
| New Jersey | 114 | 20.1 | 141 | 24.9 | 1.0 (0.73,1.4) | 1.3 (0.87,1.9) | 145 | 25.6 | 1.1 (0.77,1.5) | 1.7 (1.1,2.5) | 166 | 29.3 | 0.97 (0.70,1.3) | **1.7 (1.1,2.6)** |
| New York | 147 | 28.1 | 125 | 23.9 | **0.70 (0.50,0.97)** | 0.80 (0.55,1.2) | 139 | 26.5 | 0.80 (0.58,1.1) | 1.1 (0.75,1.7) | 113 | 21.6 | **0.51 (0.37,0.71)** | 1.0 (0.65,1.6) |
| Georgia | 137 | 22.5 | 135 | 22.1 | 0.81 (0.58,1.1) | 0.99 (0.67,1.5) | 159 | 26.1 | 0.98 (0.71,1.3) | 1.5 (0.97,2.2) | 179 | 29.3 | 0.87 (0.64,1.2) | 1.5 (0.93,2.3) |
| North Carolina | 76 | 26.0 | 73 | 25.0 | 0.79 (0.53,1.2) | 0.97 (0.62,1.5) | 66 | 22.6 | 0.73 (0.49,1.1) | 1.1 (0.64,1.8) | 77 | 26.4 | **0.68 (0.46,0.99)** | 1.3 (0.76,2.2) |
| Utah | 83 | 32.2 | 73 | 28.3 | 0.72 (0.49,1.1) | **0.60 (0.38,0.93)** | 56 | 21.7 | **0.57 (0.38,0.85)** | **0.51 (0.31,0.84)** | 46 | 17.8 | **0.37 (0.24,0.56)** | **0.51 (0.29,0.90)** |
| **Dietary fat intake (% of daily calories)** | | |  |  |  |  |  |  |  |  |  |  |  |  |
| < 30% | 844 | 26.2 | 773 | 24.0 | 1.0 | 1.0 | 786 | 24.4 | 1.0 | 1.0 | 825 | 25.6 | 1.0 | 1.0 |
| >30% | 609 | 23.6 | 679 | 26.3 | **1.2 (1.1,1.4)** | **1.5 (1.3,1.8)** | 666 | 25.8 | 1.2 (1.0,1.4) | **1.8 (1.5,2.2)** | 627 | 24.3 | **1.1 (0.91,1.2)** | **2.5 (2.0,3.0)** |
| **Pre-pregnancy Body Mass Index** | |  |  |  |  |  |  |  |  |  |  |  |  |  |
| 18.5-<25 (Normal) | 843 | 26.9 | 772 | 24.6 | 1.0 | 1.0 | 772 | 24.6 | 1.0 | 1.0 | 749 | 23.9 | 1.0 | 1.0 |
| <18.5 (Underweight) | 72 | 22.9 | 80 | 25.5 | 1.2 (0.87,1.7) | 0.83 (0.58,1.2) | 78 | 24.8 | 1.2 (0.85,1.7) | 0.97 (0.66,1.4) | 84 | 26.8 | 1.3 (0.94,1.8) | 1.1 (0.73,1.7) |
| 25 <30 (Overweight) | 301 | 24.2 | 327 | 26.3 | 1.2 (0.99,1.4) | 1.0 (0.70,1.5) | 323 | 26.0 | 1.2 (0.97,1.4) | 1.2 (0.80,1.8) | 293 | 23.6 | 1.1 (0.91,1.3) | 1.3 (0.80,2.0) |
| >30 (Obese) | 203 | 22.9 | 231 | 26.0 | 1.2 (1.0,1.5) | 1.1 (0.76,1.7) | 213 | 24.0 | 1.1 (0.92,1.4) | 1.3 (0.83,2.0) | 240 | 27.1 | **1.3 (1.1,1.6)** | **1.8 (1.1,2.9)** |
| **Folic Acid Containing Supplements (B1-P3)^c^** | | |  |  |  |  |  |  |  |  |  |  |  |  |
| no | 646 | 24.9 | 606 | 23.3 | 1.0 | 1.0 | 625 | 24.1 | 1.0 | 1.0 | 722 | 722.0 | 1.0 | 1.0 |
| yes | 799 | 25.1 | 837 | 26.3 | 1.1 (0.96,1.3) | 1.1 (0.89,1.3) | 820 | 25.8 | 1.0 (0.92,1.2) | 1.1 (0.92,1.3) | 722 | 22.7 | 0.81 (0.70,0.94) | 1.1 (0.89,1.3) |
| ^a^Referent category is total nitrite intake at the lowest quartile of total nitrite intake (<2.63578 mg/day) | | | | | | |  |  |  |  |  |  |  |  |
| ^b^Adjusted for tertiles of daily caloric intake, race/ethnicity, maternal education, state of residence, dietary fat intake, pre-pregnancy body mass index, and folic acid supplementation.  ^c^Any use one month before conception to three months after conception | | | | | | | | | | | | | |  |

|  | | | | | | | | | | | | | | | |  | |
| --- | --- | --- | --- | --- | --- | --- | --- | --- | --- | --- | --- | --- | --- | --- | --- | --- | --- |
|  | **Table F1D: Odds ratios and 95% confidence intervals for total dietary nitrosamine intake (µg/day) by maternal characteristics** | | | | | | | | | | | | | | | |  |
|  |  | **Quartile 1^a^  0.00889-0.33299** | | **Quartile 2 0.33300-0.47158** | | **Odds Ratios and 95% confidence intervals** | | **Quartile 3 0.47159-0.66846** | | **Odds Ratios and 95% confidence intervals** | | **Quartile 4**  **0.66847-7.30426** | | **Odds Ratios and 95% confidence intervals** | | |  |
|  |  | **n** | **%** | **n** | **%** | **Crude** | **Adjusted^b^** | **n** | **%** | **Crude** | **Adjusted^b^** | **n** | **%** | **Crude** | **Adjusted^b^** | |  |
|  | **Race/Ethnicity** |  |  |  |  |  |  |  |  |  |  |  |  |  |  | |  |
|  | White non-Hispanic | 761 | 21.8 | 894 | 25.7 | 1.0 | 1.0 | 917 | 26.3 | 1.0 | 1.0 | 912 | 26.2 | 1.0 | 1.0 | |  |
|  | Black non-Hispanic | 203 | 30.8 | 158 | 23.9 | **0.66 (0.53,0.83)** | **0.68 (0.53,0.88)** | 152 | 23 | **0.62 (0.49,0.78)** | **0.48 (0.36,0.63)** | 147 | 22.3 | **0.60 (0.48,0.76)** | **0.36 (0.26,0.50)** | |  |
|  | Hispanic | 357 | 27.5 | 315 | 24.2 | **0.75 (0.63,0.90)** | 0.88 (0.69,1.1) | 318 | 24.5 | **0.74 (0.62,0.88)** | **0.54 (0.41,0.71)** | 310 | 23.9 | **0.73 (0.61,0.87)** | **0.35 (0.26,0.48)** | |  |
|  | Asian/Pacific Islander | 68 | 40.2 | 32 | 18.9 | **0.40 (0.26,0.62)** | **0.37 (0.24,0.58)** | 29 | 17.2 | **0.36 (0.23,0.55)** | **0.31 (0.19,0.52)** | 40 | 23.7 | **0.49 (0.33,0.73)** | **0.33 (0.19,0.57)** | |  |
|  | Other | 53 | 31.6 | 45 | 26.8 | 0.72 (0.48,1.1) | 0.86 (0.55,1.33) | 34 | 20.2 | **0.53 (0.34,0.83)** | **0.50 (0.29,0.84)** | 36 | 21.4 | **0.57 (0.37,0.87)** | **0.42 (0.24,0.74)** | |  |
|  | **Maternal education (years completed)** | | |  |  |  |  |  |  |  |  |  |  |  |  | |  |
|  | 16+ | 402 | 22 | 508 | 27.8 | 1.0 | 1.0 | 485 | 26.6 | 1.0 | 1.0 | 431 | 23.6 | 1.0 | 1.0 | |  |
|  | 13-15 | 343 | 22.3 | 384 | 24.9 | 0.89 (0.73,1.1) | 0.90 (0.73,1.1) | 404 | 26.2 | **0.98 (0.81,1.2)** | 0.79 (0.63,0.99) | 409 | 26.6 | 1.1 (0.91,1.4) | **0.74 (0.58,0.96)** | |  |
|  | 12 | 404 | 28 | 356 | 24.7 | **0.70 (0.57,0.85)** | **0.72 (0.58,0.89)** | 330 | 22.9 | **0.68 (0.56,0.82)** | **0.46 (0.36,0.58)** | 354 | 24.5 | **0.82 (0.67,0.99)** | **0.38 (0.29,0.50)** | |  |
|  | 9-11 | 209 | 30 | 143 | 20.6 | **0.54 (0.42,0.70)** | **0.57 (0.43,0.75)** | 161 | 23.1 | **0.64 (0.50,0.82)** | **0.39 (0.29,0.53)** | 183 | 26.3 | 0.82 (0.64,1.0) | **0.33 (0.24,0.47)** | |  |
|  | 0-8 | 83 | 29.9 | 57 | 20.5 | **0.54 (0.38,0.78)** | **0.57 (0.38,0.86)** | 67 | 24.1 | **0.67 (0.47,0.95)** | **0.47 (0.30,0.73)** | 71 | 25.5 | 0.80 (0.57,1.1) | **0.37 (0.23,0.61)** | |  |
|  | **State/Area of residence** |  |  |  |  |  |  |  |  |  |  |  |  |  |  | |  |
|  | Texas | 168 | 24.6 | 166 | 24.3 | 1.0 | 1.0 | 176 | 25.7 | 1.0 | 1.0 | 174 | 25.4 | 1.0 | 1.0 | |  |
|  | Arkansas | 151 | 21.4 | 150 | 21.2 | 1.0 (0.74,1.4) | 1.0 (0.72,1.5) | 193 | 27.3 | 1.2 (0.90,1.6) | 1.1 (0.73,1.5) | 213 | 30.1 | 1.4 (1.0,1.8) | 1.0 (0.69,1.6) | |  |
|  | California | 235 | 30.1 | 174 | 22.3 | 0.75 (0.56,1.0) | 0.80 (0.59,1.1) | 171 | 27.9 | **0.69 (0.52,0.93)** | **0.65 (0.47,0.90)** | 202 | 25.8 | 0.83 (0.63,1.1) | 0.73 (0.51,1.0) | |  |
|  | Iowa | 100 | 15.5 | 118 | 18.3 | **1.2 (0.85,1.7)** | 1.2 (0.84,1.8) | 168 | 26.1 | **1.6 (1.2,2.2)** | **1.9 (1.3,2.8)** | 258 | 40.1 | **2.5 (1.8,3.4)** | **3.6 (2.4,5.5)** | |  |
|  | Massachusetts | 208 | 28.0 | 226 | 30.4 | 1.1 (0.83,1.5) | 1.0 (0.72,1.4) | 191 | 25.7 | 0.88 (0.66,1.2) | 0.88 (0.61,1.3) | 118 | 15.9 | **0.55 (0.40,0.75)** | 0.66 (0.44,1.0) | |  |
|  | New Jersey | 141 | 25.0 | 160 | 28.4 | 1.1 (0.84,1.6) | 1.1 (0.78,1.5) | 141 | 25.0 | 0.95 (0.70,1.3) | 1.0 (0.70,1.5) | 122 | 21.6 | 0.84 (0.61,1.2) | 0.87 (0.57,1.3) | |  |
|  | New York | 135 | 26.0 | 156 | 30.1 | **1.2 (0.85,1.6)** | 1.1 (0.76,1.5) | 123 | 23.7 | 0.87 (0.63,1.2) | 0.90 (0.61,1.3) | 105 | 20.2 | **0.75 (0.54,1.0)** | 0.82 (0.53,1.3) | |  |
|  | Georgia | 185 | 30.1 | 158 | 25.7 | 0.86 (0.64,1.2) | 0.88 (0.62,1.2) | 151 | 24.6 | 0.78 (0.58,1.1) | 0.83 (0.57,1.2) | 120 | 19.5 | **0.63 (0.46,0.86)** | 0.67 (0.44,1.0) | |  |
|  | North Carolina | 84 | 29.0 | 87 | 30.0 | 1.0 (0.73,1.5) | 0.99 (0.66,1.5) | 64 | 22.1 | 0.73 (0.49,1.1) | 0.64 (0.40,1.0) | 55 | 19.0 | **0.63 (0.42,0.94)** | **0.49 (0.29,0.83)** | |  |
|  | Utah | 44 | 17.2 | 56 | 21.9 | 1.3 (0.82,2.0) | 1.2 (0.71,1.9) | 73 | 28.5 | 1.6 (1.0,2.4) | 1.4 (0.84,2.3) | 83 | 32.4 | **1.8 (1.2,2.8)** | 1.8 (1.0,3.0) | |  |
|  | **Dietary fat intake (% of daily calories)** | | |  |  |  |  |  |  |  |  |  |  |  |  | |  |
|  | < 30% | 1034 | 31.9 | 871 | 26.9 | 1.0 | 1.0 | 726 | 22.4 | 1.0 | 1.0 | 606 | 18.7 | 1.0 | 1.0 | |  |
|  | >30% | 417 | 16.3 | 580 | 22.6 | **1.7 (1.4,1.9)** | **2.0 (1.7,2.4)** | 725 | 28.3 | **2.5 (2.1,2.9)** | **4.3 (3.6,5.2)** | 844 | 3.29 | **3.5 (3.0,4.0)** | **8.5 (6.9,10.4)** | |  |
|  | **Dietary folate equivalent (µg/day)** | |  |  |  |  |  |  |  |  |  |  |  |  |  | |  |
|  | <319 | 619 | 43.1 | 386 | 26.8 | 1.0 | 1.0 | 273 | 19.0 | 1.0 | 1.0 | 160 | 11.1 | 1.0 | 1.0 | |  |
|  | 319-464.9 | 362 | 24.9 | 414 | 28.5 | **1.8 (1.5,2.2)** | **2.0 (1.7,2.5)** | 395 | 27.2 | **2.5 (2.0,3.0)** | **1.8 (1.5,2.3)** | 281 | 19.4 | **3.0 (2.4,3.8)** | **1.7 (1.3,2.3)** | |  |
|  | 465-685.5 | 288 | 19.7 | 368 | 25.2 | **2.0 (1.7,2.5)** | **2.6 (2.1,3.3)** | 416 | 28.5 | **3.3 (2.7,4.0)** | **1.9 (1.5,2.5)** | 390 | 26.7 | **5.2 (4.2,6.6)** | **2.0 (1.5,2.7)** | |  |
|  | >685.5 | 182 | 12.5 | 283 | 19.5 | **2.5 (2.0,3.1)** | **3.8 (2.9,4.8)** | 367 | 25.3 | **4.6 (3.6,5.7)** | **2.0 (1.5,2.7)** | 619 | 42.7 | **13.2 (10.4,16.7)** | **3.4 (2.4,4.7)** | |  |
|  | ^a^Referent category is nitrosamine intake at the lowest quartile of nitrosamine intake (<0.33 µg/day) | | | | | | |  |  |  |  |  |  |  |  | |  |
|  | ^b^Adjusted for tertiles of daily caloric intake, race/ethnicity, maternal education, state of residence, dietary fat intake, and dietary folate as dietary folate equivalents. | | | | | | | | | | | | | |  | |  |
