# Supplementary material for: Maternal characteristics associated with the dietary intake of nitrates, nitrites, and nitrosamines in women of child-bearing age: a cross-sectional study
Source: Environ Health. 2010 Feb 19;9:10. doi: 10.1186/1476-069X-9-10 (PMC2848640; doi:10.1186/1476-069X-9-10)
Supplement: Additional file 2 — Contributions of food groups to nitrate, nitrite, total nitrite, and nitrosamine intake. These tables show the estimated contribution and percent contribution of food groups to nitrate, nitrite, total nitrite, and nitrosamine intake by race/ethnicity. [file 1476-069X-9-10-S2.DOC]

Additional File 2

Contributions of Food Groups to Nitrate, Nitrite, Total Nitrite, and Nitrosamine Intake

**Table F2A:** **1997-2004 NBDPS control mothers average daily nitrate, nitrite, and total nitrite intake**

|  | | **Vegetable Products** | | | **Fruit Products** | | | **Dairy Products** | | | **Grain Products** | | | **Meat Products** | | | **Bean Products** | | | **Fats, Oils, Nuts, and Sweets** | | |
| --- | --- | --- | --- | --- | --- | --- | --- | --- | --- | --- | --- | --- | --- | --- | --- | --- | --- | --- | --- | --- | --- | --- |
| **Nitrate** | | **n** | **mg/day** | **%** | **n** | **mg/day** | **%** | **n** | **mg/day** | **%** | **n** | **mg/day** | **%** | **n** | **mg/day** | **%** | **n** | **mg/day** | **%** | **n** | **mg/day** | **%** |
| White non-Hispanic | | 3522 | 28.327 | 62.2 | 3523 | 8.181 | 18.0 | 3525 | 1.194 | 2.6 | 3523 | 1.263 | 2.8 | 3522 | 5.000 | 11.0 | 3526 | 0.433 | 1.0 | 3515 | 1.128 | 2.5 |
| Black non-Hispanic | | 667 | 48.139 | 70.2 | 666 | 9.321 | 13.6 | 669 | 0.753 | 1.1 | 668 | 1.395 | 2.0 | 667 | 6.750 | 9.8 | 672 | 0.447 | 0.7 | 664 | 1.727 | 2.5 |
| Hispanic | | 1314 | 28.431 | 49.9 | 1316 | 16.710 | 29.3 | 1316 | 0.869 | 1.5 | 1319 | 1.897 | 3.3 | 1314 | 6.227 | 10.9 | 1322 | 1.504 | 2.6 | 1314 | 1.393 | 2.4 |
| Asian/Pacific Islander | | 172 | 64.396 | 73.8 | 173 | 11.950 | 13.7 | 172 | 0.911 | 1.0 | 173 | 2.931 | 3.4 | 174 | 5.432 | 6.2 | 174 | 0.684 | 0.8 | 173 | 1.004 | 1.2 |
| Other | | 176 | 36.090 | 60.0 | 179 | 13.141 | 21.8 | 181 | 0.944 | 1.6 | 176 | 1.774 | 2.9 | 177 | 5.731 | 9.5 | 179 | 0.903 | 1.5 | 176 | 1.574 | 2.6 |
| Missing | | 22 | 35.877 | 60.5 | 21 | 14.729 | 24.8 | 22 | 0.915 | 1.5 | 22 | 1.746 | 2.9 | 22 | 3.711 | 6.3 | 22 | 1.065 | 1.8 | 22 | 1.258 | 2.1 |
| All women | | 5873 | 31.917 | 60.9 | 5878 | 10.505 | 20.0 | 5885 | 1.054 | 2.0 | 5881 | 1.486 | 2.8 | 5876 | 5.505 | 10.5 | 5895 | 0.699 | 1.3 | 5864 | 1.265 | 2.4 |
| **Nitrite** | | **n** | **mg/day** | **%** | **n** | **mg/day** | **%** | **n** | **mg/day** | **%** | **n** | **mg/day** | **%** | **n** | **mg/day** | **%** | **n** | **mg/day** | **%** | **n** | **mg/day** | **%** |
| White non-Hispanic | | 3522 | 0.181 | 11.6 | 3523 | 0.066 | 4.3 | 3525 | 0.048 | 3.1 | 3523 | 0.177 | 11.4 | 3522 | 0.990 | 63.8 | 3526 | 0.060 | 3.9 | 3515 | 0.031 | 2.0 |
| Black non-Hispanic | | 667 | 0.216 | 11.3 | 666 | 0.061 | 3.2 | 669 | 0.040 | 2.1 | 668 | 0.200 | 10.5 | 667 | 1.300 | 67.9 | 672 | 0.049 | 2.6 | 664 | 0.048 | 2.5 |
| Hispanic | | 1314 | 0.204 | 8.8 | 1316 | 0.140 | 6.0 | 1316 | 0.049 | 2.1 | 1319 | 0.257 | 11.1 | 1314 | 1.241 | 53.5 | 1322 | 0.385 | 16.6 | 1314 | 0.043 | 1.9 |
| Asian/Pacific Islander | | 172 | 0.258 | 12.1 | 173 | 0.086 | 4.0 | 172 | 0.051 | 2.4 | 173 | 0.394 | 18.5 | 174 | 1.197 | 56.4 | 174 | 0.109 | 5.1 | 173 | 0.030 | 1.4 |
| Other | | 176 | 0.214 | 11.0 | 179 | 0.095 | 4.9 | 181 | 0.049 | 2.5 | 176 | 0.241 | 12.5 | 177 | 1.097 | 56.6 | 179 | 0.194 | 10.0 | 176 | 0.046 | 2.4 |
| Missing | | 22 | 0.219 | 12.9 | 21 | 0.105 | 6.2 | 22 | 0.049 | 2.9 | 22 | 0.248 | 14.6 | 22 | 0.856 | 50.3 | 22 | 0.182 | 10.7 | 22 | 0.044 | 2.6 |
| All women | | 5873 | 0.193 | 10.8 | 5878 | 0.084 | 4.7 | 5885 | 0.047 | 2.6 | 5881 | 0.206 | 11.5 | 5876 | 1.090 | 60.7 | 5895 | 0.138 | 7.7 | 5864 | 0.036 | 2.0 |
| **Total nitrite** | | **n** | **mg/day** | **%** | **n** | **mg/day** | **%** | **n** | **mg/day** | **%** | **n** | **mg/day** | **%** | **n** | **mg/day** | **%** | **n** | **mg/day** | **%** | **n** | **mg/day** | **%** |
| White non-Hispanic | | 3522 | 1.597 | 41.7 | 3523 | 0.475 | 12.4 | 3525 | 0.107 | 2.8 | 3523 | 0.240 | 6.3 | 3522 | 1.241 | 32.4 | 3526 | 0.082 | 2.1 | 3515 | 0.088 | 2.3 |
| Black non-Hispanic | | 667 | 2.623 | 49.2 | 666 | 0.527 | 9.9 | 669 | 0.078 | 1.5 | 668 | 0.270 | 5.1 | 667 | 1.638 | 30.7 | 672 | 0.072 | 1.3 | 664 | 0.135 | 2.5 |
| Hispanic | | 1314 | 1.625 | 31.4 | 1316 | 0.975 | 18.9 | 1316 | 0.092 | 1.8 | 1319 | 0.352 | 6.8 | 1314 | 1.552 | 30.0 | 1322 | 0.461 | 8.9 | 1314 | 0.113 | 2.2 |
| Asian/Pacific Islander | | 172 | 3.477 | 53.6 | 173 | 0.683 | 10.5 | 172 | 0.096 | 1.5 | 173 | 0.540 | 8.3 | 174 | 1.470 | 22.7 | 174 | 0.143 | 2.2 | 173 | 0.080 | 1.2 |
| Other | | 176 | 2.018 | 40.8 | 179 | 0.752 | 15.2 | 181 | 0.096 | 1.9 | 176 | 0.330 | 6.7 | 177 | 1.384 | 28.0 | 179 | 0.239 | 4.8 | 176 | 0.125 | 2.5 |
| Missing | | 22 | 2.013 | 43.1 | 21 | 0.841 | 18.0 | 22 | 0.095 | 2.0 | 22 | 0.335 | 7.2 | 22 | 1.041 | 22.3 | 22 | 0.235 | 5.0 | 22 | 0.107 | 2.3 |
| All women | | 5873 | 1.789 | 40.5 | 5878 | 0.609 | 13.8 | 5885 | 0.100 | 2.3 | 5881 | 0.281 | 6.4 | 5876 | 1.366 | 30.9 | 5895 | 0.173 | 3.9 | 5864 | 0.100 | 2.3 |
|  |  | | | | | | | | | | | | | | | | | | | | | |

NBDPS = National Birth Defects Prevention Study

**Table F2B: 1997-2004 NBDPS control mothers average daily nitrosamine intake**

| **Nitrosamine^a^** | **Vegetable Products** | | |  | **Fruit Products** | | |  | **Dairy Products** | | |  | **Grain Products** | | |  | **Meat Products** | | |  | **Fats, Oils, Nuts, and Sweets** | | |  | **Alcohol** | | |
| --- | --- | --- | --- | --- | --- | --- | --- | --- | --- | --- | --- | --- | --- | --- | --- | --- | --- | --- | --- | --- | --- | --- | --- | --- | --- | --- | --- |
|  | **n** | **µg/day** | **%** |  | **n** | **µg/day** | **%** |  | **n** | **µg/day** | **%** |  | **n** | **µg/day** | **%** |  | **n** | **µg/day** | **%** |  | **n** | **µg/day** | **%** |  | **n** | **µg/day** | **%** |
| White non-Hispanic | 3522 | 0.000 | 0.0 |  | 3523 | 0.008 | 1.4 |  | 3525 | 0.269 | 49.0 |  | 3523 | 0.010 | 1.8 |  | 3519 | 0.242 | 44.2 |  | 3515 | 0.007 | 1.2 |  | 3507 | 0.013 | 2.4 |
| Black non-Hispanic | 667 | 0.000 | 0.0 |  | 666 | 0.009 | 1.6 |  | 669 | 0.159 | 29.7 |  | 668 | 0.014 | 2.6 |  | 667 | 0.325 | 60.8 |  | 664 | 0.007 | 1.3 |  | 669 | 0.021 | 3.9 |
| Hispanic | 1314 | 0.000 | 0.0 |  | 1316 | 0.013 | 2.5 |  | 1316 | 0.202 | 37.4 |  | 1319 | 0.015 | 2.8 |  | 1314 | 0.294 | 54.5 |  | 1314 | 0.005 | 1.0 |  | 1320 | 0.01 | 1.9 |
| Asian/Pacific Islander | 172 | 0.000 | 0.0 |  | 173 | 0.012 | 2.3 |  | 172 | 0.193 | 38.0 |  | 173 | 0.008 | 1.5 |  | 174 | 0.290 | 57.1 |  | 173 | 0.004 | 0.9 |  | 173 | 0.001 | 0.2 |
| Other | 176 | 0.000 | 0.0 |  | 179 | 0.012 | 2.3 |  | 181 | 0.212 | 42.1 |  | 176 | 0.012 | 2.4 |  | 177 | 0.256 | 50.8 |  | 176 | 0.006 | 1.2 |  | 170 | 0.006 | 1.2 |
| Missing | 22 | 0.000 | 0.0 |  | 21 | 0.014 | 3.5 |  | 22 | 0.190 | 45.5 |  | 22 | 0.013 | 3.2 |  | 22 | 0.195 | 46.7 |  | 22 | 0.005 | 1.1 |  | 22 | 0.000 | 0.0 |
| All women | 5873 | 0.000 | 0.0 |  | 5878 | 0.009 | 1.7 |  | 5885 | 0.237 | 43.7 |  | 5881 | 0.012 | 2.1 |  | 5873 | 0.265 | 48.9 |  | 5864 | 0.006 | 1.2 |  | 5861 | 0.013 | 2.4 |

^a^Bean products were estimated with a nitrosamine content of o.ooo **µg** and are, therefore, not subcategorized.

NBDPS = National Birth Defects Prevention Study
