# Supplementary material for: Maternal characteristics associated with the dietary intake of nitrates, nitrites, and nitrosamines in women of child-bearing age: a cross-sectional study
Source: Environ Health. 2010 Feb 19;9:10. doi: 10.1186/1476-069X-9-10 (PMC2848640; doi:10.1186/1476-069X-9-10)
Supplement: Additional file 3 — Contributions of individual food items to average daily nitrite, nitrite and nitrosamine intake by race/ethnicity. This table shows the average nitrate, nitrite, and nitrosamine contribution per day from each food item on the National Birth Defects Prevention Study food frequency questionnaire by race/ethnicity. [file 1476-069X-9-10-S3.DOC]

Additional File 3

Contributions of Individual Food Items to Average Daily Nitrate, Nitrite, and Nitrosamine Intake by Race/ethnicity

| **Table F3a: Contributions of food items to average daily nitrate, nitrite, and nitrosamine intake by race/ethnicity** | | | | | | | | | | | | | | | | | | | | | | | | |
| --- | --- | --- | --- | --- | --- | --- | --- | --- | --- | --- | --- | --- | --- | --- | --- | --- | --- | --- | --- | --- | --- | --- | --- | --- |
|  | **White non-Hispanic** | | | | | | **Black non-Hispanic** | | | | | | **Hispanic** | | | | | | **Asian/Pacific Islander** | | | | | |
|  |
| ***Food Item*** | **n** | | **Average daily intake** | | **Average servings**  **per day** | | **n** | | **Average daily intake** | | **Average servings**  **per day** | | **n** | | **Average daily intake** | | **Average servings**  **per day** | | **n** | | **Average daily intake** | | **Average servings**  **per day** | |
| ***Estimated nitrate contribution in mg/day from food item*** | | | | | | | | | | | | | | | | | | | | | | | | |
|  |  | | **mg/day**  **nitrate** | |  | |  | | **mg/day**  **nitrate** | |  | |  | | **mg/day**  **nitrate** | |  | |  | | **mg/day**  **nitrate** | |  | |
| **Skim or low fat milk (8 oz glass)** | 3531 | | 0.76 | | 0.877^b^ | | 673 | | 0.27 | | 0.317 | | 1321 | | 0.33 | | 0.389 | | 175 | | 0.53 | | 0.612^b^ | |
| **Whole milk (8 oz glass)** | 3532 | | 0.05 | | 0.238 | | 673 | | 0.13 | | 0.622^b^ | | 1322 | | 0.15 | | 0.730^b^ | | 175 | | 0.09 | | 0.421 | |
| **Yogurt ( 1 cup)** | 3532 | | 0.07 | | 0.218 | | 673 | | 0.06 | | 0.177 | | 1323 | | 0.10 | | 0.300 | | 175 | | 0.14 | | 0.411 | |
| **Ice cream (1/2 cup)** | 3530 | | 0.05 | | 0.220 | | 670 | | 0.06 | | 0.286 | | 1324 | | 0.07 | | 0.314 | | 174 | | 0.05 | | 0.220 | |
| **Cottage or Ricotta cheese (1/2 cup)** | 3531 | | 0.02 | | 0.083 | | 672 | | 0.01 | | 0.023 | | 1324 | | 0.03 | | 0.128 | | 174 | | 0.01 | | 0.054 | |
| **Other cheese e.g. American, cheddar, etc. plain or part of a dish (1 slice or 1 oz serving)** | 3529 | | 0.25 | | 0.620^b^ | | 672 | | 0.22 | | 0.556 | | 1321 | | 0.19 | | 0.464 | | 174 | | 0.10 | | 0.253 | |
| **Margarine (pat), added to food or bread** | 3527 | | 0.00 | | 0.419 | | 672 | | 0.00 | | 0.462 | | 1322 | | 0.00 | | 0.249 | | 175 | | 0.00 | | 0.202 | |
| **Butter (pat), added to food or bread** | 3528 | | 0.00 | | 0.329 | | 673 | | 0.00 | | 0.316 | | 1321 | | 0.00 | | 0.260 | | 175 | | 0.00 | | 0.204 | |
| **Fresh apples or pears (1)** | 3529 | | 0.47 | | 0.329 | | 671 | | 0.60 | | 0.415 | | 1322 | | 0.86 | | 0.599^b^ | | 175 | | 0.83 | | 0.581^b^ | |
| **Oranges (1)** | 3529 | | 0.70 | | 0.191 | | 671 | | 1.38 | | 0.375 | | 1322 | | 2.10 | | 0.570 | | 175 | | 1.73 | | 0.471 | |
| **Orange juice (small glass)** | 3530 | | 1.70^a^ | | 0.456^b^ | | 672 | | 2.30^a^ | | 0.619^b^ | | 1321 | | 2.63^a^ | | 0.708^b^ | | 175 | | 2.41 | | 0.648^b^ | |
| **Peaches, apricots, plums, or nectarines (1 fresh or 1/2 cup canned)** | 3530 | | 0.09 | | 0.156 | | 672 | | 0.14 | | 0.247 | | 1322 | | 0.19 | | 0.332 | | 175 | | 0.17 | | 0.300 | |
| **Bananas (1)** | 3531 | | 0.79 | | 0.346 | | 672 | | 0.87 | | 0.380 | | 1322 | | 1.21 | | 0.531 | | 175 | | 1.13 | | 0.496 | |
| **Other fruits fresh, frozen, or canned (1/2 cup)** | 3530 | | 1.06 | | 0.284 | | 671 | | 1.02 | | 0.275 | | 1319 | | 1.07 | | 0.287 | | 175 | | 1.29 | | 0.346 | |
| **Tomatoes (1) or tomato juice (small glass)** | 3531 | | 1.09 | | 0.276 | | 673 | | 0.82 | | 0.207 | | 1322 | | 1.78 | | 0.450 | | 174 | | 1.48 | | 0.375 | |
| **String beans (1/2 cup)** | 3531 | | 2.06^a^ | | 0.175 | | 671 | | 2.56^a^ | | 0.217 | | 1321 | | 1.69 | | 0.144 | | 174 | | 2.10 | | 0.178 | |
| **Broccoli (1/2 cup)** | 3531 | | 4.19^a^ | | 0.158 | | 671 | | 4.43^a^ | | 0.167 | | 1322 | | 5.23^a^ | | 0.196 | | 174 | | 6.39^a^ | | 0.240 | |
| **Cabbage, cauliflower, or Brussel sprouts (1/2 cup)** | 3528 | | 0.75 | | 0.058 | |  | | 1.53 | | 0.118 | | 1322 | | 1.76 | | 0.136 | | 174 | | 2.93^a^ | | 0.226 | |
| **Carrots, raw (1/2 cup or 2-4 sticks)** | 3530 | | 1.27 | | 0.211 | | 671 | | 0.83 | | 0.139 | | 1321 | | 1.48 | | 0.246 | | 172 | | 1.49 | | 0.248 | |
| **Carrots, cooked (1/2 cup)** | 3529 | | 0.98 | | 0.075 | | 672 | | 0.97 | | 0.075 | | 1322 | | 1.88 | | 0.145 | | 172 | | 2.19 | | 0.168 | |
| **Corn (1 ear or 1/2 cup frozen, canned)** | 3530 | | 0.76 | | 0.205 | | 671 | | 0.94 | | 0.256 | | 1321 | | 0.93 | | 0.252 | | 174 | | 0.81 | | 0.220 | |
| **Peas or lima beans (1/2 cup frozen, canned)** | 3529 | | 0.25 | | 0.095 | | 672 | | 0.30 | | 0.115 | | 1322 | | 0.19 | | 0.071 | | 174 | | 0.34 | | 0.129 | |
| **Yams or sweet potatoes (1/2 cup)** | 3531 | | 0.09 | | 0.030 | | 672 | | 0.29 | | 0.093 | | 1321 | | 0.16 | | 0.053 | | 174 | | 0.21^a^ | | 0.066 | |
| **Spinach or collard greens, cooked (1/2 cup)** | 3531 | | 10.79^a^ | | 0.057 | | 670 | | 30.45^a^ | | 0.161 | | 1322 | | 9.27^a^ | | 0.049 | | 174 | | 41.96 | | 0.222 | |
| **Beans or lentils, baked or dried (1/2 cup)** | 3529 | | 0.07 | | 0.062 | | 672 | | 0.09 | | 0.075 | | 1322 | | 0.31 | | 0.263 | | 174 | | 0.23 | | 0.194 | |
| **Squash (1/2 cup)** | 3530 | | 1.57 | | 0.036 | | 671 | | 1.11 | | 0.026 | | 1322 | | 1.76 | | 0.040 | | 174 | | 2.13 | | 0.049 | |
| **Eggs (1)** | 3531 | | 0.09 | | 0.294 | | 669 | | 0.18 | | 0.582^b^ | | 1321 | | 0.15 | | 0.486 | | 174 | | 0.13 | | 0.416 | |
| **Chicken or turkey with skin (4-6 oz)** | 3529 | | 0.23 | | 0.395 | | 669 | | 0.29 | | 0.498 | | 1321 | | 0.22 | | 0.370 | | 174 | | 0.24 | | 0.411 | |
| **Bacon (2 slices)** | 3529 | | 0.13 | | 0.090 | | 668 | | 0.39 | | 0.272 | | 1322 | | 0.16 | | 0.112 | | 174 | | 0.10 | | 0.070 | |
| **Hot dogs (1)** | 3530 | | 0.77 | | 0.067 | | 669 | | 1.51 | | 0.130 | | 1322 | | 0.93 | | 0.081 | | 174 | | 0.68 | | 0.059 | |
| **Processed meats, e.g. sausage, salami, bologna, chorizo, etc (piece or slice)** | 3529 | | 0.18 | | 0.108 | | 668 | | 0.40 | | 0.237 | | 1320 | | 0.36 | | 0.209 | | 174 | | 0.10 | | 0.061 | |
| **Liver (3-4 oz)** | 3530 | | 0.02 | | 0.002 | | 670 | | 0.26 | | 0.025 | | 1321 | | 0.28 | | 0.027 | | 174 | | 0.26 | | 0.025 | |
| **Hamburger (1 patty)** | 3530 | | 1.04 | | 0.171 | | 670 | | 1.34 | | 0.219 | | 1321 | | 1.06 | | 0.174 | | 174 | | 0.63 | | 0.103 | |
| **Beef, pork, lamb or cabrito as a sandwich or mixed dish, e.g. stew, casserole, lasagna, etc** | 3529 | | 1.00 | | 0.171 | | 670 | | 0.88 | | 0.151 | | 1320 | | 1.08 | | 0.184 | | 174 | | 1.48 | | 0.252 | |
| **Beef, pork, lamb or cabrito as a main dish, e.g. steak, roast, ham, etc (4-6 oz)** | 3527 | | 1.44 | | 0.175 | | 670 | | 1.31 | | 0.160 | | 1318 | | 1.83 | | 0.224 | | 174 | | 1.55 | | 0.190 | |
| **Fish (3-6 oz)** | 3530 | | 0.09 | | 0.088 | | 669 | | 0.18 | | 0.174 | | 1321 | | 0.12 | | 0.113 | | 174 | | 0.21 | | 0.208 | |
| **Chocolate (1 oz)** | 3529 | | 0.00 | | 0.436 | | 669 | | 0.00 | | 0.382 | | 1321 | | 0.00 | | 0.310 | | 174 | | 0.00 | | 0.230 | |
| **Candy without chocolate (1 oz)** | 3529 | | 0.00 | | 0.193 | | 669 | | 0.00 | | 0.291 | | 1321 | | 0.00 | | 0.181 | | 174 | | 0.00 | | 0.177 | |
| **Pie (slice)** | 3529 | | 0.09 | | 0.037 | | 670 | | 0.17 | | 0.073 | | 1320 | | 0.23 | | 0.097 | | 174 | | 0.11 | | 0.045 | |
| **Cake (slice) or donut (1)** | 3528 | | 0.09 | | 0.055 | | 670 | | 0.16 | | 0.100 | | 1320 | | 0.13 | | 0.080 | | 174 | | 0.09 | | 0.059 | |
| **Cookies (1)** | 3528 | | 0.03 | | 0.274 | | 668 | | 0.03 | | 0.305 | | 1320 | | 0.03 | | 0.348 | | 174 | | 0.03 | | 0.279 | |
| **White bread (slice), including pita bread, bagels and crackers** | 3529 | | 0.24 | | 0.590^b^ | | 670 | | 0.31 | | 0.759^b^ | | 1320 | | 0.19 | | 0.454 | | 174 | | 0.18 | | 0.431 | |
| **Dark bread (slice) including wheat pita bread** | 3527 | | 0.09 | | 0.445 | | 669 | | 0.07 | | 0.347 | | 1319 | | 0.06 | | 0.303 | | 174 | | 0.09 | | 0.419 | |
| **French fried potatoes (4 oz)** | 3529 | | 0.71 | | 0.170 | | 669 | | 1.02 | | 0.247 | | 1320 | | 0.79 | | 0.189 | | 174 | | 0.63 | | 0.151 | |
| **Potatoes baked, boiled (1) or mashed (1 cup)** | 3527 | | 5.87^a^ | | 0.259 | | 669 | | 5.01^a^ | | 0.221 | | 1317 | | 4.39^a^ | | 0.194 | | 174 | | 3.64^a^ | | 0.161 | |
| **Rice or pasta, e.g. Spanish rice, spaghetti, noodles, etc (1 cup)** | 3527 | | 0.78 | | 0.336 | | 668 | | 0.83 | | 0.361 | | 1320 | | 1.07 | | 0.464 | | 174 | | 2.53^a^ | | 1.098^b^ | |
| **Potato chips or corn chips (small bag or 1 oz)** | 3528 | | 0.20 | | 0.188 | | 668 | | 0.32 | | 0.303 | | 1319 | | 0.21 | | 0.199 | | 174 | | 0.13 | | 0.122 | |
| **Nuts (small packet or 1 oz)** | 3528 | | 0.01 | | 0.079 | | 668 | | 0.01 | | 0.075 | | 1319 | | 0.01 | | 0.059 | | 174 | | 0.01 | | 0.119 | |
| **Peanut butter (1 tbs)** | 3528 | | 0.02 | | 0.201 | | 669 | | 0.01 | | 0.134 | | 1320 | | 0.01 | | 0.110 | | 174 | | 0.01 | | 0.099 | |
| **Oil and vinegar dressing e.g. Italian (1 tbs)** | 3529 | | 0.00 | | 0.171 | | 669 | | 0.00 | | 0.121 | | 1320 | | 0.00 | | 0.101 | | 173 | | 0.00 | | 0.181 | |
| **Cantaloupe (1/4 melon)** | 3529 | | 1.13 | | 0.089 | | 672 | | 1.53 | | 0.120 | | 1322 | | 2.36 | | 0.186 | | 175 | | 1.91 | | 0.150 | |
| **Avocado (1) or guacamole (1 cup)** | 3531 | | 0.17 | | 0.031 | | 673 | | 0.11 | | 0.020 | | 1321 | | 0.99 | | 0.188 | | 175 | | 0.24 | | 0.045 | |
| **Raw chile peppers, jalapeño (1)** | 3530 | | 0.03 | | 0.039 | | 671 | | 0.05 | | 0.072 | | 1322 | | 0.31 | | 0.413 | | 174 | | 0.24 | | 0.324 | |
| **Salsa (1 cup) (fruit or tomato)** | 3531 | | 0.95 | | 0.105 | | 670 | | 0.51 | | 0.056 | | 1321 | | 3.26^a^ | | 0.359 | | 173 | | 0.56 | | 0.061 | |
| **Chicken Livers (1 oz)** | 3530 | | 0.00 | | 0.002 | | 670 | | 0.02 | | 0.017 | | 1321 | | 0.02 | | 0.016 | | 174 | | 0.02 | | 0.018 | |
| **Organ meats barbacoa, menudo, sweetbreads, tongue, intestines (3-4 oz)** | 3530 | | 0.00 | | 0.001 | | 670 | | 0.01 | | 0.006 | | 1321 | | 0.05 | | 0.057 | | 174 | | 0.02 | | 0.027 | |
| **Tortilla (1)** | 3527 | | 0.03 | | 0.110 | | 669 | | 0.01 | | 0.042 | | 1320 | | 0.42 | | 1.364**^b^** | | 173 | | 0.04 | | 0.145 | |
| **Refried beans (1 cup)** | 3530 | | 0.11 | | 0.049 | | 672 | | 0.05 | | 0.024 | | 1322 | | 1.01 | | 0.444 | | 174 | | 0.12 | | 0.052 | |
| **Cereal** | 3543 | | 0.12 | | 0.849^b^ | | 679 | | 0.17 | | 1.199^b^ | | 1328 | | 0.16 | | 1.181^b^ | | 176 | | 0.09 | | 0.642^b^ | |
| ***Estimated nitrite contribution in mg/day from food item*** | | | | | | | | | | | | | | | | | | | | | | | | |
|  |  | | **mg/day**  **nitrite** | |  | |  | | **mg/day**  **nitrite** | |  | |  | | **mg/day**  **nitrite** | |  | |  | | **mg/day**  **nitrite** | |  | |
| **Skim or low fat milk (8 oz glass)** | 3531 | | 0.01 | | 0.877^b^ | | 673 | | 0.00 | | 0.317 | | 1321 | | 0.00 | | 0.389 | | 175 | | 0.01 | | 0.612^b^ | |
| **Whole milk (8 oz glass)** | 3532 | | 0.00 | | 0.238 | | 673 | | 0.00 | | 0.622^b^ | | 1322 | | 0.00 | | 0.730^b^ | | 175 | | 0.00 | | 0.421 | |
| **Yogurt ( 1 cup)** | 3532 | | 0.02 | | 0.218 | | 673 | | 0.01 | | 0.177 | | 1323 | | 0.02 | | 0.300 | | 175 | | 0.03 | | 0.411 | |
| **Ice cream (1/2 cup)** | 3530 | | 0.01 | | 0.220 | | 670 | | 0.01 | | 0.286 | | 1324 | | 0.01 | | 0.314 | | 174 | | 0.01 | | 0.220 | |
| **Cottage or Ricotta cheese (1/2 cup)** | 3532 | | 0.00 | | 0.083 | | 673 | | 0.00 | | 0.023 | | 1324 | | 0.00 | | 0.128 | | 175 | | 0.00 | | 0.054 | |
| **Other cheese e.g. American, cheddar, etc. plain or part of a dish (1 slice or 1 oz serving)** | 3529 | | 0.01 | | 0.620^b^ | | 672 | | 0.01 | | 0.556 | | 1321 | | 0.01 | | 0.464 | | 174 | | 0.01 | | 0.253 | |
| **Margarine (pat), added to food or bread** | 3529 | | 0.00 | | 0.419 | | 672 | | 0.00 | | 0.462 | | 1322 | | 0.00 | | 0.249 | | 175 | | 0.00 | | 0.202 | |
| **Butter (pat), added to food or bread** | 3525 | | 0.00 | | 0.329 | | 671 | | 0.00 | | 0.316 | | 1321 | | 0.00 | | 0.260 | | 175 | | 0.00 | | 0.204 | |
| **Fresh apples or pears (1)** | 3529 | | 0.00 | | 0.329 | | 671 | | 0.01 | | 0.415 | | 1322 | | 0.01 | | 0.599^b^ | | 175 | | 0.01 | | 0.581^b^ | |
| **Oranges (1)** | 3530 | | 0.00 | | 0.191 | | 671 | | 0.00 | | 0.375 | | 1322 | | 0.00 | | 0.570 | | 175 | | 0.00 | | 0.471 | |
| **Orange juice (small glass)** | 3530 | | 0.00 | | 0.456^b^ | | 672 | | 0.00 | | 0.619^b^ | | 1321 | | 0.00 | | 0.708^b^ | | 175 | | 0.00 | | 0.648^b^ | |
| **Peaches, apricots, plums, or nectarines (1 fresh or 1/2 cup canned)** | 3530 | | 0.00 | | 0.156 | | 672 | | 0.00 | | 0.247 | | 1322 | | 0.00 | | 0.332 | | 175 | | 0.00 | | 0.300 | |
| **Bananas (1)** | 3531 | | 0.02 | | 0.346 | | 672 | | 0.02 | | 0.380 | | 1322 | | 0.02 | | 0.531 | | 175 | | 0.02 | | 0.496 | |
| **Other fruits fresh, frozen, or canned (1/2 cup)** | 3530 | | 0.01 | | 0.284 | | 671 | | 0.01 | | 0.275 | | 1319 | | 0.01 | | 0.287 | | 175 | | 0.01 | | 0.346 | |
| **Tomatoes (1) or tomato juice (small glass)** | 3531 | | 0.01 | | 0.276 | | 673 | | 0.01 | | 0.207 | | 1322 | | 0.02 | | 0.450 | | 174 | | 0.02 | | 0.375 | |
| **String beans (1/2 cup)** | 3531 | | 0.02 | | 0.175 | | 671 | | 0.03 | | 0.217 | | 1321 | | 0.02 | | 0.144 | | 174 | | 0.02 | | 0.178 | |
| **Broccoli (1/2 cup)** | 3531 | | 0.04 | | 0.158 | | 671 | | 0.04 | | 0.167 | | 1322 | | 0.05 | | 0.196 | | 174 | | 0.06 | | 0.240 | |
| **Cabbage, cauliflower, or Brussel sprouts (1/2 cup)** | 3528 | | 0.01 | | 0.058 | | 672 | | 0.02 | | 0.118 | | 1322 | | 0.02 | | 0.136 | | 174 | | 0.03 | | 0.226 | |
| **Carrots, raw (1/2 cup or 2-4 sticks)** | 3530 | | 0.01 | | 0.211 | | 671 | | 0.01 | | 0.139 | | 1321 | | 0.01 | | 0.246 | | 172 | | 0.01 | | 0.248 | |
| **Carrots, cooked (1/2 cup)** | 3529 | | 0.01 | | 0.075 | | 672 | | 0.01 | | 0.075 | | 1322 | | 0.02 | | 0.145 | | 172 | | 0.02 | | 0.168 | |
| **Corn (1 ear or 1/2 cup frozen, canned)** | 3530 | | 0.03 | | 0.205 | | 671 | | 0.04 | | 0.256 | | 1321 | | 0.04 | | 0.252 | | 174 | | 0.04 | | 0.220 | |
| **Peas or lima beans (1/2 cup frozen, canned)** | 3529 | | 0.01 | | 0.095 | | 672 | | 0.01 | | 0.115 | | 1322 | | 0.01 | | 0.071 | | 174 | | 0.01 | | 0.129 | |
| **Yams or sweet potatoes (1/2 cup)** | 3531 | | 0.00 | | 0.030 | | 672 | | 0.00 | | 0.093 | | 1321 | | 0.00 | | 0.053 | | 174 | | 0.00 | | 0.066 | |
| **Spinach or collard greens, cooked (1/2 cup)** | 3531 | | 0.01 | | 0.057 | | 670 | | 0.03 | | 0.161 | | 1322 | | 0.01 | | 0.049 | | 174 | | 0.04 | | 0.222 | |
| **Beans or lentils, baked or dried (1/2 cup)** | 3529 | | 0.02 | | 0.062 | | 672 | | 0.03 | | 0.075 | | 1322 | | 0.09 | | 0.263 | | 174 | | 0.07 | | 0.194 | |
| **Squash (1/2 cup)** | 3530 | | 0.00 | | 0.036 | | 671 | | 0.00 | | 0.026 | | 1322 | | 0.00 | | 0.040 | | 174 | | 0.00 | | 0.049 | |
| **Eggs (1)** | 3531 | | 0.03 | | 0.294 | | 669 | | 0.06 | | 0.582^b^ | | 1321 | | 0.05 | | 0.486 | | 174 | | 0.04 | | 0.416 | |
| **Chicken or turkey with skin (4-6 oz)** | 3529 | | 0.19^a^ | | 0.395 | | 669 | | 0.24^a^ | | 0.498 | | 1321 | | 0.18^a^ | | 0.370 | | 174 | | 0.20^a^ | | 0.411 | |
| **Bacon (2 slices)** | 3529 | | 0.04 | | 0.090 | | 668 | | 0.13^a^ | | 0.272 | | 1322 | | 0.05 | | 0.112 | | 174 | | 0.03 | | 0.070 | |
| **Hot dogs (1)** | 3530 | | 0.08^a^ | | 0.067 | | 669 | | 0.16^a^ | | 0.130 | | 1322 | | 0.10 | | 0.081 | | 174 | | 0.07 | | 0.059 | |
| **Processed meats, e.g. sausage, salami, bologna, chorizo, etc (piece or slice)** | 3529 | | 0.03 | | 0.108 | | 668 | | 0.07 | | 0.237 | | 1320 | | 0.07 | | 0.209 | | 174 | | 0.02 | | 0.061 | |
| **Liver (3-4 oz)** | 3530 | | 0.00 | | 0.002 | | 670 | | 0.04 | | 0.025 | | 1321 | | 0.04 | | 0.027 | | 174 | | 0.04 | | 0.025 | |
| **Hamburger (1 patty)** | 3530 | | 0.03 | | 0.171 | | 670 | | 0.04 | | 0.219 | | 1321 | | 0.03 | | 0.174 | | 174 | | 0.02 | | 0.103 | |
| **Beef, pork, lamb or cabrito as a sandwich or mixed dish, e.g. stew, casserole, lasagna, etc** | 3529 | | 0.22^a^ | | 0.171 | | 670 | | 0.20^a^ | | 0.151 | | 1320 | | 0.24^a^ | | 0.184 | | 174 | | 0.33^a^ | | 0.252 | |
| **Beef, pork, lamb or cabrito as a main dish, e.g. steak, roast, ham, etc (4-6 oz)** | 3527 | | 0.32^a^ | | 0.175 | | 670 | | 0.29^a^ | | 0.160 | | 1318 | | 0.41^a^ | | 0.224 | | 174 | | 0.35^a^ | | 0.190 | |
| **Fish (3-6 oz)** | 3530 | | 0.03 | | 0.088 | | 669 | | 0.06 | | 0.174 | | 1321 | | 0.04 | | 0.113 | | 174 | | 0.07^a^ | | 0.208 | |
| **Chocolate (1 oz)** | 3528 | | 0.00 | | 0.436 | | 669 | | 0.00 | | 0.382 | | 1321 | | 0.00 | | 0.310 | | 174 | | 0.00 | | 0.230 | |
| **Candy without chocolate (1 oz)** | 3527 | | 0.00 | | 0.193 | | 669 | | 0.00 | | 0.291 | | 1320 | | 0.00 | | 0.181 | | 174 | | 0.00 | | 0.177 | |
| **Pie (slice)** | 3529 | | 0.01 | | 0.037 | | 670 | | 0.01 | | 0.073 | | 1320 | | 0.01 | | 0.097 | | 174 | | 0.01 | | 0.045 | |
| **Cake (slice) or donut (1)** | 3528 | | 0.00 | | 0.055 | | 670 | | 0.01 | | 0.100 | | 1320 | | 0.01 | | 0.080 | | 174 | | 0.00 | | 0.059 | |
| **Cookies (1)** | 3528 | | 0.00 | | 0.274 | | 668 | | 0.00 | | 0.305 | | 1320 | | 0.00 | | 0.348 | | 174 | | 0.00 | | 0.279 | |
| **White bread (slice), including pita bread, bagels and crackers** | 3529 | | 0.02 | | 0.590^b^ | | 670 | | 0.03 | | 0.759^b^ | | 1320 | | 0.02 | | 0.454 | | 174 | | 0.02 | | 0.431 | |
| **Dark bread (slice) including wheat pita bread** | 3527 | | 0.01 | | 0.445 | | 669 | | 0.01 | | 0.347 | | 1319 | | 0.01 | | 0.303 | | 174 | | 0.01 | | 0.419 | |
| **French fried potatoes (4 oz)** | 3529 | | 0.01 | | 0.170 | | 669 | | 0.02 | | 0.247 | | 1320 | | 0.01 | | 0.189 | | 174 | | 0.01 | | 0.151 | |
| **Potatoes baked, boiled (1) or mashed (1 cup)** | 3527 | | 0.04 | | 0.259 | | 669 | | 0.04 | | 0.221 | | 1317 | | 0.03 | | 0.194 | | 174 | | 0.03 | | 0.161 | |
| **Rice or pasta, e.g. Spanish rice, spaghetti, noodles, etc (1 cup)** | 3527 | | 0.10^a^ | | 0.336 | | 668 | | 0.11 | | 0.361 | | 1320 | | 0.14^a^ | | 0.464 | | 174 | | 0.33^a^ | | 1.098^b^ | |
| **Potato chips or corn chips (small bag or 1 oz)** | 3528 | | 0.00 | | 0.188 | | 668 | | 0.01 | | 0.303 | | 1319 | | 0.00 | | 0.199 | | 174 | | 0.00 | | 0.122 | |
| **Nuts (small packet or 1 oz)** | 3528 | | 0.00 | | 0.079 | | 668 | | 0.00 | | 0.075 | | 1319 | | 0.00 | | 0.059 | | 174 | | 0.00 | | 0.119 | |
| **Peanut butter (1 tbs)** | 3528 | | 0.00 | | 0.201 | | 669 | | 0.00 | | 0.134 | | 1320 | | 0.00 | | 0.110 | | 174 | | 0.00 | | 0.099 | |
| **Oil and vinegar dressing e.g. Italian (1 tbs)** | 3529 | | 0.00 | | 0.171 | | 669 | | 0.00 | | 0.121 | | 1320 | | 0.00 | | 0.101 | | 173 | | 0.00 | | 0.181 | |
| **Cantaloupe (1/4 melon)** | 3529 | | 0.00 | | 0.089 | | 672 | | 0.01 | | 0.120 | | 1322 | | 0.01 | | 0.186 | | 175 | | 0.01 | | 0.150 | |
| **Avocado (1) or guacamole (1 cup)** | 3531 | | 0.00 | | 0.031 | | 673 | | 0.00 | | 0.020 | | 1321 | | 0.01 | | 0.188 | | 175 | | 0.00 | | 0.045 | |
| **Raw chile peppers, jalapeño (1)** | 3530 | | 0.00 | | 0.039 | | 671 | | 0.00 | | 0.072 | | 1322 | | 0.00 | | 0.413 | | 174 | | 0.00 | | 0.324 | |
| **Salsa (1 cup) (fruit or tomato)** | 3531 | | 0.01 | | 0.105 | | 670 | | 0.01 | | 0.056 | | 1321 | | 0.05 | | 0.359 | | 173 | | 0.01 | | 0.061 | |
| **Chicken Livers (1 oz)** | 3530 | | 0.00 | | 0.002 | | 670 | | 0.01 | | 0.017 | | 1321 | | 0.01 | | 0.016 | | 174 | | 0.01 | | 0.018 | |
| **Organ meats barbacoa, menudo, sweetbreads, tongue, intestines (3-4 oz)** | 3530 | | 0.00 | | 0.001 | | 670 | | 0.00 | | 0.006 | | 1321 | | 0.03 | | 0.057 | | 174 | | 0.01 | | 0.027 | |
| **Tortilla (1)** | 3527 | | 0.00 | | 0.110 | | 669 | | 0.00 | | 0.042 | | 1320 | | 0.04 | | 1.364^b^ | | 173 | | 0.00 | | 0.145 | |
| **Refried beans (1 cup)** | 3530 | | 0.03 | | 0.049 | | 672 | | 0.02 | | 0.024 | | 1322 | | 0.29^a^ | | 0.444 | | 174 | | 0.03 | | 0.052 | |
| **Cereal** | 3543 | | 0.03 | | 0.849^b^ | | 679 | | 0.05 | | 1.199^b^ | | 1328 | | 0.05 | | 1.181^b^ | | 176 | | 0.03 | | 0.642^a^ | |
| ***Estimated nitrosamine contribution in µg/day from food item*** | | | | | | | | | | | | | | | | | | | | | | | | |
|  | |  | | **µg/day**  **nitrosamine** | |  | |  | | **µg/day**  **nitrosamine** | |  | |  | | **µg/day**  **nitrosamine** | |  | |  | | **µg/day**  **nitrosamine** | |  |
| **Skim or low fat milk (8 oz glass)** | 3531 | | 0.184^a^ | | 0.877^b^ | | 673 | | 0.066^a^ | | 0.317 | | 1321 | | 0.081^a^ | | 0.389 | | 175 | | 0.128^a^ | | 0.612^b^ | |
| **Whole milk (8 oz glass)** | 3532 | | 0.015 | | 0.238 | | 673 | | 0.040 | | 0.622^b^ | | 1322 | | 0.047^a^ | | 0.730^b^ | | 175 | | 0.027 | | 0.421 | |
| **Yogurt ( 1 cup)** | 3532 | | 0.001 | | 0.218 | | 673 | | 0.000 | | 0.177 | | 1323 | | 0.001 | | 0.300 | | 175 | | 0.001 | | 0.411 | |
| **Ice cream (1/2 cup)** | 3530 | | 0.007 | | 0.220 | | 670 | | 0.009 | | 0.286 | | 1324 | | 0.010 | | 0.314 | | 174 | | 0.007 | | 0.220 | |
| **Cottage or Ricotta cheese (1/2 cup)** | 3531 | | 0.022 | | 0.083 | | 672 | | 0.006 | | 0.023 | | 1324 | | 0.034 | | 0.128 | | 174 | | 0.014 | | 0.054 | |
| **Other cheese e.g American, cheddar, etc. plain or part of a dish (1 slice or 1 oz serving)** | 3529 | | 0.041^a^ | | 0.620^b^ | | 672 | | 0.037 | | 0.556 | | 1321 | | 0.031 | | 0.464 | | 174 | | 0.017 | | 0.253 | |
| **Margarine (pat), added to food or bread** | 3530 | | 0.000 | | 0.419 | | 672 | | 0.000 | | 0.462 | | 1322 | | 0.000 | | 0.249 | | 175 | | 0.000 | | 0.202 | |
| **Butter (pat), added to food or bread** | 3529 | | 0.000 | | 0.329 | | 673 | | 0.000 | | 0.316 | | 1321 | | 0.000 | | 0.260 | | 175 | | 0.000 | | 0.204 | |
| **Fresh apples or pears (1)** | 3529 | | 0.002 | | 0.329 | | 671 | | 0.003 | | 0.415 | | 1322 | | 0.004 | | 0.599^b^ | | 175 | | 0.004 | | 0.581^b^ | |
| **Oranges (1)** | 3530 | | 0.000 | | 0.191 | | 671 | | 0.000 | | 0.375 | | 1322 | | 0.000 | | 0.570 | | 175 | | 0.000 | | 0.471 | |
| **Orange juice (small glass)** | 3530 | | 0.000 | | 0.456^b^ | | 672 | | 0.000 | | 0.619^b^ | | 1321 | | 0.000 | | 0.708^b^ | | 175 | | 0.000 | | 0.648^b^ | |
| **Peaches, apricots, plums, or nectarines (1 fresh or 1/2 cup canned)** | 3530 | | 0.001 | | 0.156 | | 672 | | 0.001 | | 0.247 | | 1322 | | 0.001 | | 0.332 | | 175 | | 0.001 | | 0.300 | |
| **Bananas (1)** | 3531 | | 0.002 | | 0.346 | | 672 | | 0.002 | | 0.380 | | 1322 | | 0.003 | | 0.531 | | 175 | | 0.003 | | 0.496 | |
| **Other fruits fresh, frozen, or canned (1/2 cup)** | 3530 | | 0.002 | | 0.284 | | 671 | | 0.002 | | 0.275 | | 1319 | | 0.002 | | 0.287 | | 175 | | 0.002 | | 0.346 | |
| **Tomatoes (1) or tomato juice (small glass)** | 3531 | | 0.000 | | 0.276 | | 673 | | 0.000 | | 0.207 | | 1322 | | 0.000 | | 0.450 | | 174 | | 0.000 | | 0.375 | |
| **String beans (1/2 cup)** | 3531 | | 0.000 | | 0.175 | | 671 | | 0.000 | | 0.217 | | 1321 | | 0.000 | | 0.144 | | 174 | | 0.000 | | 0.178 | |
| **Broccoli (1/2 cup)** | 3531 | | 0.000 | | 0.158 | | 672 | | 0.000 | | 0.167 | | 1322 | | 0.000 | | 0.196 | | 174 | | 0.000 | | 0.240 | |
| **Cabbage, cauliflower, or Brussel sprouts (1/2 cup)** | 3530 | | 0.000 | | 0.058 | | 672 | | 0.000 | | 0.118 | | 1322 | | 0.000 | | 0.136 | | 174 | | 0.000 | | 0.226 | |
| **Carrots, raw (1/2 cup or 2-4 sticks)** | 3531 | | 0.000 | | 0.211 | | 671 | | 0.000 | | 0.139 | | 1321 | | 0.000 | | 0.246 | | 174 | | 0.000 | | 0.248 | |
| **Carrots, cooked (1/2 cup)** | 3530 | | 0.000 | | 0.075 | | 672 | | 0.000 | | 0.075 | | 1322 | | 0.000 | | 0.145 | | 174 | | 0.000 | | 0.168 | |
| **Corn (1 ear or 1/2 cup frozen, canned)** | 3530 | | 0.000 | | 0.205 | | 671 | | 0.000 | | 0.256 | | 1322 | | 0.000 | | 0.252 | | 174 | | 0.000 | | 0.220 | |
| **Peas or lima beans (1/2 cup frozen, canned)** | 3530 | | 0.000 | | 0.095 | | 672 | | 0.000 | | 0.115 | | 1322 | | 0.000 | | 0.071 | | 174 | | 0.000 | | 0.129 | |
| **Yams or sweet potatoes (1/2 cup)** | 3531 | | 0.000 | | 0.030 | | 672 | | 0.000 | | 0.093 | | 1322 | | 0.000 | | 0.053 | | 174 | | 0.000 | | 0.066 | |
| **Spinach or collard greens, cooked (1/2 cup)** | 3531 | | 0.000 | | 0.057 | | 671 | | 0.000 | | 0.161 | | 1322 | | 0.000 | | 0.049 | | 174 | | 0.000 | | 0.222 | |
| **Beans or lentils, baked or dried (1/2 cup)** | 3531 | | 0.000 | | 0.062 | | 672 | | 0.000 | | 0.075 | | 1322 | | 0.000 | | 0.263 | | 174 | | 0.000 | | 0.194 | |
| **Squash (1/2 cup)** | 3531 | | 0.000 | | 0.036 | | 671 | | 0.000 | | 0.026 | | 1322 | | 0.000 | | 0.040 | | 174 | | 0.000 | | 0.049 | |
| **Eggs (1)** | 3531 | | 0.000 | | 0.294 | | 669 | | 0.000 | | 0.582^b^ | | 1322 | | 0.000 | | 0.486 | | 174 | | 0.000 | | 0.416 | |
| **Chicken or turkey with skin (4-6 oz)** | 3529 | | 0.034 | | 0.395 | | 669 | | 0.043 | | 0.498 | | 1321 | | 0.032 | | 0.370 | | 174 | | 0.036 | | 0.411 | |
| **Bacon (2 slices)** | 3529 | | 0.020 | | 0.090 | | 668 | | 0.060^a^ | | 0.272 | | 1322 | | 0.024 | | 0.112 | | 174 | | 0.015 | | 0.070 | |
| **Hot dogs (1)** | 3530 | | 0.009 | | 0.067 | | 669 | | 0.017 | | 0.130 | | 1322 | | 0.010 | | 0.081 | | 174 | | 0.008 | | 0.059 | |
| **Processed meats, e.g. sausage, salami, bologna, chorizo, etc (piece or slice)** | 3529 | | 0.013 | | 0.108 | | 668 | | 0.029 | | 0.237 | | 1320 | | 0.026 | | 0.209 | | 174 | | 0.008 | | 0.061 | |
| **Liver (3-4 oz)** | 3530 | | 0.000 | | 0.002 | | 670 | | 0.001 | | 0.025 | | 1321 | | 0.001 | | 0.027 | | 174 | | 0.001 | | 0.025 | |
| **Hamburger (1 patty)** | 3530 | | 0.012 | | 0.171 | | 670 | | 0.016 | | 0.219 | | 1321 | | 0.012 | | 0.174 | | 174 | | 0.007 | | 0.103 | |
| **Beef, pork, lamb or cabrito as a sandwich or mixed dish, e.g. stew, casserole, lasagna, etc** | 3529 | | 0.055^a^ | | 0.171 | | 670 | | 0.049^a^ | | 0.151 | | 1320 | | 0.060^a^ | | 0.184 | | 174 | | 0.082^a^ | | 0.252 | |
| **Beef, pork, lamb or cabrito as a main dish, e.g. steak, roast, ham, etc (4-6 oz)** | 3527 | | 0.079^a^ | | 0.175 | | 670 | | 0.072^a^ | | 0.160 | | 1318 | | 0.101^a^ | | 0.224 | | 174 | | 0.086^a^ | | 0.190 | |
| **Fish (3-6 oz)** | 3530 | | 0.019 | | 0.088 | | 669 | | 0.039 | | 0.174 | | 1321 | | 0.025 | | 0.113 | | 174 | | 0.046^a^ | | 0.208 | |
| **Chocolate (1 oz)** | 3528 | | 0.004 | | 0.436 | | 669 | | 0.004 | | 0.382 | | 1321 | | 0.003 | | 0.310 | | 174 | | 0.002 | | 0.230 | |
| **Candy without chocolate (1 oz)** | 3527 | | 0.002 | | 0.193 | | 669 | | 0.003 | | 0.291 | | 1318 | | 0.002 | | 0.181 | | 174 | | 0.002 | | 0.177 | |
| **Pie (slice)** | 3529 | | 0.000 | | 0.037 | | 670 | | 0.000 | | 0.073 | | 1320 | | 0.000 | | 0.097 | | 174 | | 0.000 | | 0.045 | |
| **Cake (slice) or donut (1)** | 3529 | | 0.000 | | 0.055 | | 670 | | 0.000 | | 0.100 | | 1320 | | 0.000 | | 0.080 | | 174 | | 0.000 | | 0.059 | |
| **Cookies (1)** | 3528 | | 0.000 | | 0.274 | | 668 | | 0.001 | | 0.305 | | 1320 | | 0.001 | | 0.348 | | 174 | | 0.000 | | 0.279 | |
| **White bread (slice), including pita bread, bagels and crackers** | 3529 | | 0.001 | | 0.590^b^ | | 670 | | 0.002 | | 0.759^b^ | | 1320 | | 0.001 | | 0.454 | | 174 | | 0.001 | | 0.431 | |
| **Dark bread (slice) including wheat pita bread** | 3529 | | 0.000 | | 0.445 | | 669 | | 0.000 | | 0.347 | | 1320 | | 0.000 | | 0.303 | | 174 | | 0.000 | | 0.419 | |
| **French fried potatoes (4 oz)** | 3529 | | 0.000 | | 0.170 | | 669 | | 0.000 | | 0.247 | | 1320 | | 0.000 | | 0.189 | | 174 | | 0.000 | | 0.151 | |
| **Potatoes baked, boiled (1) or mashed (1 cup)** | 3528 | | 0.000 | | 0.259 | | 669 | | 0.000 | | 0.221 | | 1320 | | 0.000 | | 0.194 | | 174 | | 0.000 | | 0.161 | |
| **Rice or pasta, e.g. Spanish rice, spaghetti, noodles, etc (1 cup)** | 3527 | | 0.000 | | 0.336 | | 668 | | 0.000 | | 0.361 | | 1320 | | 0.000 | | 0.464 | | 174 | | 0.000 | | 1.098^b^ | |
| **Potato chips or corn chips (small bag or 1 oz)** | 3528 | | 0.000 | | 0.188 | | 668 | | 0.000 | | 0.303 | | 1320 | | 0.000 | | 0.199 | | 174 | | 0.000 | | 0.122 | |
| **Nuts (small packet or 1 oz)** | 3529 | | 0.000 | | 0.079 | | 668 | | 0.000 | | 0.075 | | 1320 | | 0.000 | | 0.059 | | 174 | | 0.000 | | 0.119 | |
| **Peanut butter (1 tbs)** | 3528 | | 0.000 | | 0.201 | | 669 | | 0.000 | | 0.134 | | 1320 | | 0.000 | | 0.110 | | 174 | | 0.000 | | 0.099 | |
| **Oil and vinegar dressing e.g. Italian (1 tbs)** | 3529 | | 0.000 | | 0.171 | | 669 | | 0.000 | | 0.121 | | 1320 | | 0.000 | | 0.101 | | 173 | | 0.000 | | 0.181 | |
| **Cantaloupe (1/4 melon)** | 3529 | | 0.001 | | 0.089 | | 672 | | 0.001 | | 0.120 | | 1322 | | 0.001 | | 0.186 | | 175 | | 0.001 | | 0.150 | |
| **Avocado (1) or guacamole (1 cup)** | 3531 | | 0.000 | | 0.031 | | 673 | | 0.000 | | 0.020 | | 1321 | | 0.002 | | 0.188 | | 175 | | 0.000 | | 0.045 | |
| **Raw chile peppers, jalapeño (1)** | 3531 | | 0.000 | | 0.039 | | 671 | | 0.000 | | 0.072 | | 1322 | | 0.000 | | 0.413 | | 174 | | 0.000 | | 0.324 | |
| **Salsa (1 cup) (fruit or tomato)** | 3531 | | 0.000 | | 0.105 | | 670 | | 0.000 | | 0.056 | | 1321 | | 0.000 | | 0.359 | | 173 | | 0.000 | | 0.061 | |
| **Chicken Livers (1 oz)** | 3530 | | 0.000 | | 0.002 | | 670 | | 0.000 | | 0.017 | | 1321 | | 0.000 | | 0.016 | | 174 | | 0.000 | | 0.018 | |
| **Organ meats barbacoa, menudo, sweetbreads, tongue, intestines (3-4 oz)** | 3530 | | 0.000 | | 0.001 | | 670 | | 0.000 | | 0.006 | | 1321 | | 0.004 | | 0.057 | | 174 | | 0.002 | | 0.027 | |
| **Tortilla (1)** | 3527 | | 0.000 | | 0.110 | | 669 | | 0.000 | | 0.042 | | 1320 | | 0.002 | | 1.364^b^ | | 173 | | 0.000 | | 0.145 | |
| **Refried beans (1 cup)** | 3530 | | 0.000 | | 0.049 | | 672 | | 0.000 | | 0.024 | | 1322 | | 0.000 | | 0.444 | | 174 | | 0.000 | | 0.052 | |
| **Cereal** | 3507 | | 0.536^a^ | | 0.849^b^ | | 661 | | 0.512^a^ | | 1.199^b^ | | 1302 | | 0.528^a^ | | 1.181^b^ | | 170 | | 0.500^a^ | | 0.642^b^ | |

^a^ Represents one of the top five food items based on contribution to total daily intake. The top five items represent differences in food choices and are presented for each

compound (nitrate, nitrite, nitrosamines).

^b^ Represents one of the top five food items based on average number of servings per day. The top five food items represent differences in food choices and are indicated for

each compound (nitrate, nitrite, nitrosamines).
